# Permeability scaling relationships of volcanic tuff from core to field scale measurements

**DOI:** 10.1038/s41598-025-96835-5

**Published:** 2025-04-15

**Authors:** Dolan D. Lucero, S. Michelle Bourret, John P. Ortiz, Bradley G. Fritz, Miles A. Bodmer, Jason E. Heath, Kristopher L. Kuhlman, Hakim Boukhalfa, Shawn Otto, Souheil Ezzedine, Barry L. Roberts, R. Charles Choens, Mark A. Person, Philip H. Stauffer, PE1 Experimental Team, Hakim Boukhalfa, Shawn Otto, Souheil Ezzedine, George Abbott, Thomas Alexander, Ethan Alger, Adan Alvarez, Tarabay Antoun, Graham Auld, Perry Barrow, Tara Bartlett, Miles Bodmer, Kyren Bogolub, Jesse Bonner, Rose Borden, Chris Bradley, Scott Broome, Brian Brown, Jeff Burghardt, Charles Choens, Al Churby, Alexander Couture, Glenn Crosby, Alvaro Cruz-Cabrera, Walter Dekin, Matthew Dietel, Christine Downs, Nicholas Downs, Elizabeth Dzenitis, Eric Eckert, Stephanie Eras, Garrett Euler, Jose Falliner, Jim Fast, Kristine Featherston, Joshua Feldman, Michael Foxe, Clayton Freimuth, Bradley Fritz, Graham Galvin, Sergio Gamboa, Lisa Garner, Jason Gastelum, Jessie Gaylord, David Gessey, Matthew Goodwin, James Griego, Scott Grover, Dylan Hauk, Jason Heath, Austin Holand, James Holdcroft, Will Honjas, Matthew Ingraham, Johnny Jaramillo, Aryton Jenkins, Kyle Jones, Graham Kent, Michael Keogh, Will Kibikas, Hunter Knox, James Knox, Kristopher Kuhlman, Jennifer Larotonda, Dorothy Linneman, Paul Lipkowitz, Gordon MacLeod, Erin McCann, Rob Mellors, Brian Memmott, Jennifer Mendez, Xavier Miller, Manny Montano, Joseph Morris, William Munley, Dea Musa, Steve Myers, Annabelle Navarro, Rose Perea, Jacob Peterson, Gabe Plank, Mike Poskey, Matthew Powell, Amanda Price, Andrew Puyleart, Justin Reppart, Hernan Rico, Barry Roberts, Rebecca Rodd, Mark Rodriguez, Alexander Romanczuk, Melissa Roth, George Salyer, Bill Savran, Cari Seifert, Dana Sirota, Dave Slater, Devon Smith, Ken Smith, Cathy Snelson, Brady Spears, Philip Stauffer, Richard Stead, Mary Stephens, Chris Strickland, Joshua Tafoya, M.’balia Tagoe, Stephanie Teich-McGoldrick, Ben Terry, Ryan Thompson, Margaret Townsend, Greg Tubbs, Reagan Turley, Nichole Valdez, Oleg Vorobiev, Robert White, Aliya Whitehill, Marc Williams, Jennifer Wilson, Lynn Wood, Andrew Wright, Guangping Xu, Cleat Zeiler

**Affiliations:** 1 Los Alamos National Laboratory, Los Alamos, USA; 2 Pacific Northwest National Laboratory, Richland, USA; 3 Sandia National Laboratories, Albuquerque, USA; 4 Lawrence Livermore National Laboratory, Livermore, USA; 5 New Mexico Institute of Mining and Technology, Socorro, USA; 6 Atomic Weapons Establishment, Berkshire, England; 7 Nevada National Security Site, Nevada, USA; 8 Mission Support and Test Services, North Las Vegas, USA; 9 University of Nevada, Reno, USA

**Keywords:** Environmental sciences, Hydrology

## Abstract

A recent chemical explosive test in P-Tunnel at the Nevada National Security Site, Nevada, USA, was conducted to better understand how signals propagate from explosions in the subsurface. A primary signal of interest is the migration of gases that can be used to differentiate chemical from nuclear explosions. Gas migration is highly dependent on the rock permeability which is notoriously difficult to determine experimentally in the field due to a potentially large dependence on the scale over which measurements are made. Here, we present pre-explosion permeability estimates to characterize the geologic units surrounding the recent test. Permeability measurements were made at three scales of increasing size: core samples (≈2 cm), borehole packer system tests (≈1 m), and a pre-shot cavity pressurization test (> 10 m) across ten tuff units. Permeability estimates based on core measurements showed little difference from borehole packer tests. However, permeability in most rock units calibrated from cavity pressurization tests resulted in higher permeability estimates by up to two orders of magnitude. Here, we demonstrate that the scale of the measurement significantly impacts the characterization efforts of hydraulic properties in volcanic tuff, and that local-scale measurements (< 10 m scale) do not incorporate enough heterogeneity to accurately predict field-scale flow and mass transport.

## Introduction

Understanding the migration of gases following an underground nuclear explosion (UNE) is a vital part of the National Nuclear Security Administration (NNSA) mission of world-wide nuclear test monitoring^1^. UNEs produce radionuclide gases such as xenon (Xe) that can migrate to the surface minutes to months following an explosion, driven by gas migration mechanisms that include pressure and thermal forcing combined with barometric (i.e., atmospheric) pressure variations^2–10^. Detection of certain radioactive gases that have leaked into the atmosphere is considered a “smoking gun” that an explosion was nuclear, rather than chemical.

Understanding gas migration from underground explosions is improved through controlled observations of subsurface gas transport through experimentation. The Physics Experiment 1 (PE1) is series of chemical explosions (PE1-A, -B, -D_L_) designed to allow real-time monitoring of gas migration in the rocks surrounding the explosion^1^. Successful predictions and interpretation of gas transport experiments require rigorous characterization of hydraulic properties of the geologic units surrounding the blast^11,12^. Characterization of the hydraulic properties of the rocks at P-Tunnel, which houses PE1, has been done through a joint effort of multiple national laboratories^1^. PE1-A, the first experiment of the series, was a chemical explosion test performed in P-Tunnel in October 2023.

**Fig. 1:**
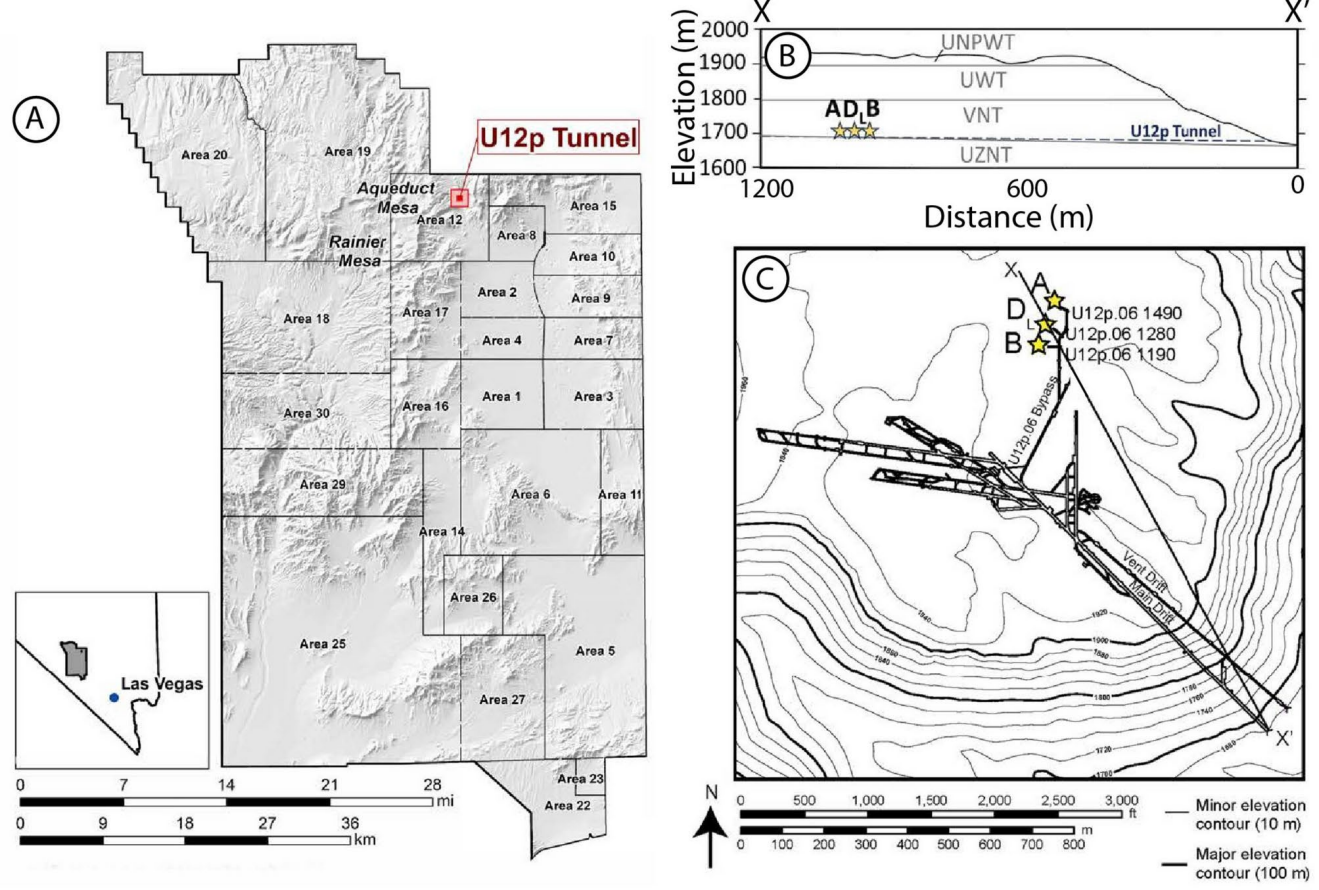
(**A**) Location of the Nevada National Security Site, Aqueduct Mesa (red box) which houses U12p Tunnel (P-Tunnel). (**B**) Cross section of Aqueduct Mesa from X – X’ in (**C**). The layers correspond to the following lithologies: upper nonwelded to partially welded tuff (UNPWT), upper welded tuff (UWT), vitric nonwelded tuff (VNT), and upper zeolitic nonwelded tuff (UZNT). (**C**) Topographic contours of Aqueduct Mesa and P-Tunnel Complex. Yellow stars represent the locations of the Physical Experiment 1 (PE1) chemical explosion experiments (PE1-A, -B, and -D_L_). Figure modified from Myers et al. (2024).

P-Tunnel is located within Aqueduct Mesa in the Nevada National Security Site (NNSS; Fig. 1)^13^. The PE1-A experiment is located within primarily volcanic tuff geology. Volcanic tuff is highly heterogeneous and its hydraulic properties are controlled by the degree of welding, presence of fractures, mineralogy, and weathering^14^. Characterization of tuff lithologies is an integral part of research into the monitoring of nuclear tests, subsurface remediation of legacy disposal sites, and siting of deep geologic nuclear waste repositories. At Los Alamos National Laboratory (LANL), legacy nuclear and hazardous waste disposal areas are located within volcanic tuff (Bandelier Tuff Formation). Previous research has relied on flow and transport characterization of volcanic tuff to analyze and simulate groundwater chromium plume migration in the aquifer beneath LANL^15,16^, volatile organic compounds (VOCs) and tritium plumes in the unsaturated zone^17–19^, radioactive contamination in soils surrounding transuranic liquid waste facility in TA-50 and -54^20^ and moisture and contaminant migration through the vadose zone from waste pits at Material Disposal Area G^21,22^. Extensive permeability and tracer transport tests have been performed to analyze the flow properties of welded and partially welded tuffs and to determine their suitability to store nuclear waste at Yucca Mountain, NV^23,24^. Additional tuff testing related to the Yucca Mountain Project was performed at the Apache Leap Research Site, Arizona, USA (ALRS)^25–28^.

Quantification of permeability depends heavily on the scale at which the experiment/simulation is performed^29^. Derived permeabilities are observed to change by orders of magnitude between measurements at the laboratory (0.01 – 10 m), borehole (10 – 100 m) and regional (1000 + m) scales^29^. There is strong evidence of scaling effects in the volcanic tuffs at the Yucca Mountain and ALRS. Cross-well permeability tests exhibit an increase in permeability of two orders of magnitude (ALRS^25–28^; Yucca Mountain^24^) compared to single-well tests. The scaling effects at the ALRS have been attributed to fractures in welded tuff observed across boreholes^30^. The orientation, density, and connectivity of fractures create a directional dependency in the scaling effects^31^. In contrast, the non-welded tuffs of the Crater Flats Group in the Yucca Mountains are zeolitized and contain few fractures, leading to a matrix-flow-dominated regime. Cross-well tests in boreholes at Yucca Mountain are thought to be influenced by a nearby fault^24^. However, within a single borehole, the scaling effect is negligible. Illman et al. (2004)^31^ incrementally increased the testing interval of a packer test within a single borehole (0.5 – 20 m) and found no distinguishable trend; permeability was within one order of magnitude. The scaling effects at the ALRS have been explained numerically using stochastic-scaling theory^32,33^ which treats the permeability field as a random fractal^34,35^. Similar scaling effects have been seen in other non-tuff geology and are highly dependent on the scale at which they are sampled and the heterogeneities the scale incorporates^36–40^.

In preparation for the PE1-A experiment, a high explosive source was emplaced in a 4 m per side rectangular cavity. After emplacement of the high explosives and backfilling the void space with sand, the access drift to the cavity was sealed by a grout plug. Gas samplers with pressure transducers were placed in boreholes 11 to 41 m away from the cavity and grouted in place. A cavity pressurization test was performed to assess the integrity of the grout plug representing the upper end of the spatial scales we used to measure permeability. Estimating the permeability field serves two primary roles: informing engineering decisions such as optimizing borehole sensor placement by way of predictive modeling, and in interpretation of the flow and transport patterns measured following the experiment. Accuracy in the permeability estimates at the relevant scale of interest is therefore critical to the success of several main goals of PE1-A. Uncertainty and heterogeneity in the vitric non-welded tuff (VNT) permeability specifically stems from several factors. Although microfractures are observed in the VNT layers^41^, the weak strength of the rock inhibits the maintenance of open fractures^14^. The VNT units lie on the boundary between the non-welded vitric tuff and altered zeolitic tuff^14^. Within this transition zone, chemical alteration has influenced the pore structure from the addition of clays and zeolites which affect permeability^42^. We hypothesize that estimated values of matrix permeability will be influenced by scale effects as the incorporation of heterogeneity increases with growing spatial scale. Much like the upscaling from single- to cross-well pumping tests in the ALRS and Yucca Mountain sites, VNT unit bulk permeability is expected to increase with the introduction of preferential flow resulting from the presence of high-permeability channels within the matrix as the length scale increases.

In this study, we estimate permeability in the PE1-A test bed at three scales. Permeabilities are estimated from mercury intrusion measurements on core samples (measurement scale ≈ 2.54 cm), within boreholes (measurement scale ≈ 1 m), and by pressurizing the pre-shot experimental cavity (measurement scale > 10 m). Core permeability was estimated using mercury intrusion. Borehole permeability was calculated using data from a dual-packer system that interrogated 0.5 to 2 m sections of boreholes by measuring the flow rate of air versus injection interval pressure. The cavity pressurization test to assess the grout plug integrity was leveraged to characterize subsurface tuff gas-phase permeability by measuring pressure changes in nearby gas sampling boreholes (11 to 41 m away) caused by pressure in the cavity. Both the permeability estimates of the packer and cavity pressurization tests rely on parameter estimation using multiphase flow and transport simulations.

## Physical and hydraulic characterization of P-tunnel geology for numerical models

The geology in which the P-Tunnel complex is situated is primarily composed of pumice- and ash-fall and ash flow deposits (Timber Mountain Formation) from the southwestern Nevada volcanic field approximately 10 km away, which erupted between 15 and 11 Ma^43^. The volcanic deposits cover the pre-eruption topography of sedimentary rock heavily deformed after a compressional event in the Cretaceous^44^. Some tuffs have been reworked and exhibit various degrees of welding and zeolitization, however, much of the volcanic lithology has experienced little structural deformation from Basin and Range Extension^45^. The P-Tunnel is approximately 350 m above the water table. Perched aquifers have been observed in other nearby tunnels, but P-Tunnel remains relatively dry^14^. Figure 1B shows the lithology of the Aqueduct Mesa, where P-Tunnel resides. From the surface to 400 m depth, the lithology is as follows: upper nonwelded to partially welded tuff (UNPWT), upper welded tuff (UWT), vitric nonwelded tuff (VNT) and upper zeolitic nonwelded tuff (UZNT). The eastern portion of P-Tunnel lies mostly within the VNT^14^. The PE1-A cavity is positioned within the transition zone where the nonwelded tuff becomes progressively zeolitized with increasing depth as it grades downward into the UZNT. Eleven geologically distinct subunits have been identified within the VNT units exposed by P-Tunnel excavation. The units are ordered from shallowest to deepest, VNT-a lies above VNT-1–10, with VNT-10 being the deepest.

Heterogeneity in tuff forms via depositional or diagenetic processes. These heterogeneities include the degree of welding, weathering, and presence of fractures all of which are present at the P-Tunnel site^14^. The degree of welding is dependent on the temperature of the ash when it was deposited. The zeolitization and clay formation occurs via weathering of tuff in events such as water table fluctuations. The presence of these minerals reduces porosity and permeability. Tuff may develop fractures or microfractures due to proximal seismic events, both anthropogenic and natural. The development is controlled by the strength of the tuff and the proximity to the seismic event. Nine fractures were identified in the drift near the PE1-A cavity with apertures less than 5 mm^12^. Depending on the type and prevalence of heterogeneous features, the scaling effects will increase or decrease the permeability of the volume of rock. Characterization studies were performed within the P-Tunnel to understand the physical and hydraulic characteristics of the VNT units. A multi-scale approach was utilized to quantify the scaling effects at three scales: 1) core/grab sample, 2) packer test, 3) cavity pressurization tests.

### VNT layer core/grab sample characterization

A series of boreholes (AC-1, GI-2, GI-3, GI-4, GI-5, HF-1, and GS-1–8) were drilled in the drifts to characterize the inter- and intra-subunit heterogeneity in the VNT. Core and grab sample analysis is described in detail in Bodmer et al. (2024)^12^. To briefly summarize, core samples from the boreholes and samples collected from the drift walls (i.e., grab samples) underwent in-situ water content analysis, thermogravimeteric analysis, and gas and mercury porosimetry to characterize their hydraulic and physical properties (saturation, density, porosity, and relative permeability)^11^. Permeabilities derived from mercury injection capillary pressure (MICP) analysis were calculated following Swanson (1981)^46^. Pore size distributions were obtained using the Young–Laplace equation and values of the surface tension of mercury and the mercury-air/vacuum-rock contact angle^11^. The porosity measurements obtained from these analyses were corroborated through petrographic observations of thin sections.

To understand the nature of unsaturated flow, Heath et al. (2021)^41^ performed mercury intrusion and direct air–water measurements on tuff core samples across the NNSS. Of the samples the authors analyzed, one is in the P-tunnel complex with similar lithology to the VNT layers (UE-12p#7, 601.5). Petrographic analysis found the pore structure is composed of a triple continuum of matrix, pre-existing and explosion-induced microfractures. Mercury intrusion and direct air–water measurements showed that this complicated pore size distribution creates a complex capillary pressure response that can be fit by a multimodal van Genutchen model and is discussed in Supplementary Information section. Macrofractures are not present in the VNT layers described as poorly fractured by Prothro et al. (2018)^14^. This is due to the relatively low strength of tuff and lack of cooling joints. Consequently, we did not consider a dual permeability/porosity model in our simulations, which can represent the effects of both fracture and matrix.

### Borehole permeability test design

The packer tests were performed in boreholes GI-2, GI-3, and GI-4 during March 2021, AC-1, GI-5, GI-6, and HF-1 during June 2022 and GS-1–8 during August to November 2022. The procedures for each packer test are explained in detail in Fritz et al. (2023)^47^. In short, the dual-packer assembly consists of two inflatable packers inserted into a borehole. Air is pumped at a constant rate into the void space between the packers, known as the injection interval. Pressure is recorded once a steady-state pressure is reached in the injection interval. Packer tests are usually performed in triplicate with varying injection rates. The steady-state interval pressure can be used to estimate the permeability of the surrounding rock, analytically or numerically.

Gas samplers were installed in the GS-1–8 boreholes. Boreholes GS-2, -4, -6, -7, and -8 were drilled from the tunnel upward into the surrounding rock, while GS-1, -3, -5 were drilled with a downward orientation. During August-November 2022, the upward-oriented holes utilized a single-packer assembly installed with the gas sampler whereas down-going boreholes have a dual-packer system. Preliminary testing of hardware, grout mixture and grouting method (in a near-by practice borehole) indicated that there was some potential for grout migration around the packer, resulting in an impact on the permeability of the sampling zone. Several measures were taken to mitigate this risk, including the use of a dual packer string on the down-going boreholes. Other measures included a thicker grout recipe close to the packer and a multi-lift grout emplacement strategy to reduce the head pressure on the liquid grout closest to the sampling zone. Packer pressurizations were performed before and after grout was emplaced behind the packers to ensure minimal disruption to the VNT permeability. In total, 105 packer tests were performed within 15 boreholes. In the AC, GI, and HF boreholes, packer tests were performed at multiple depths to characterize each of the VNT units.

A previous PE1-related investigation of gas permeability used numerical modeling to replicate experimental data from the dual-packer; pressure versus flow tests in boreholes and determined the gas permeability of eleven VNT subunits^12^. This preliminary characterization assumed constant hydraulic properties across the VNT units. Subsequent analysis of core and “grab” samples of the eleven VNT subunits showed that saturation and porosity vary significantly between units on a meter to submeter scale. Variations in saturation and porosity that were not included in the original permeability estimates require an update to the initial analysis.

### Cavity pressurization test design

Prior to the PE1-A experiment, a cavity pressurization test was performed to verify the integrity of the grout plug, sealing the drift access from the cavity. Air was pumped into the cavity (injection rate: 15,000 to 30,000 L/min) in two stages following the first and third pour of the grout plug. Five pours were conducted in total. The cavity was pressurized for 2.5 h. At the time of the pressurization test, the volume of the cavity was 32 m^3^ and was filled the explosive device and sand. Pressure transducers installed in the cavity and in the GS-1–8 boreholes recorded pressure signal at 5 to 35 m away from the center of the cavity and across 6 of the 11 VNT layers. Pressure in the cavity peaked at ~ 0.15 MPa (~ 22 psi) and transducers continued recording for several days after the pressurization stopped to observe the pressure decay proximal to the cavity. The integrity of the grout plug was successfully verified, and the pressure recorded in GS-1–8 was used to calculate the permeability of each VNT layer.

## Numerical simulations

Permeability calibration of the VNT units was achieved using numerical models and an inverse code, iteratively adjusting parameters and running the model until the data and model results reach a best fit. The porous media flow and transport simulator (FEHM)^48,49^ simulates gas flow in the packer and cavity pressurization tests. Parameter optimization is performed by the inverse code PEST^50^. PEST calibrates permeability either by the Gauss-Marquardt-Levenberg method^51^ for the packer test and Tikhonov Regularization^52^ for the cavity pressurization test. Previous investigations using FEHM coupled with PEST have been used to calibrate subsurface flow and transport properties in similar hydrologic environments including Yucca Mountain, NV and the Apache Leap Research Site, AZ^5,24,30,53^.

### Governing equations for flow and mass transport

FEHM is used to simulate multiphase isothermal porous flow through the VNT matrix. Since the permeability tests were conducted in the subsurface, temperatures are assumed to be constant and isothermal properties are used. The gas flow and injection in unsaturated porous media is given by:

1 $$\frac{{\partial \left( {\left( {\theta - 1} \right)\rho _{a} \phi } \right)}}{{\partial t}} = ~\nabla ~ \cdot \left[ {\frac{{k\rho _{a} }}{{\mu _{a} }}k\left( \theta \right)\left( {\nabla p_{a} - \rho _{a} gz} \right) + Q_{a} } \right]$$

where $\theta$ is saturation, ${\rho }_{a}$ and ${\mu }_{a}$ is the density and viscosity of air, respectively, $\phi$ is porosity, $t$ is time, $k$ is the intrinsic permeability, ${p}_{a}$ is the air pressure, $g$ is gravity, $z$ is depth, and $Qa$ is an air source term. The relative permeability of each phase, $k\left(\theta \right)$, is a function of saturation. Here, we use a multimodal van Genutchen relative permeability model based on Heath et al. (2021)^41^. The non-linearity is a result of the pore distribution, a combination of matrix and post-/pre-existing microfractures, in VNT units. All simulations are performed on a 2D mesh that assumes radial symmetry around the x-axis or y-axis in the packer and cavity pressurization tests, respectively.

### Packer test model design and assumptions

**Fig. 2:**
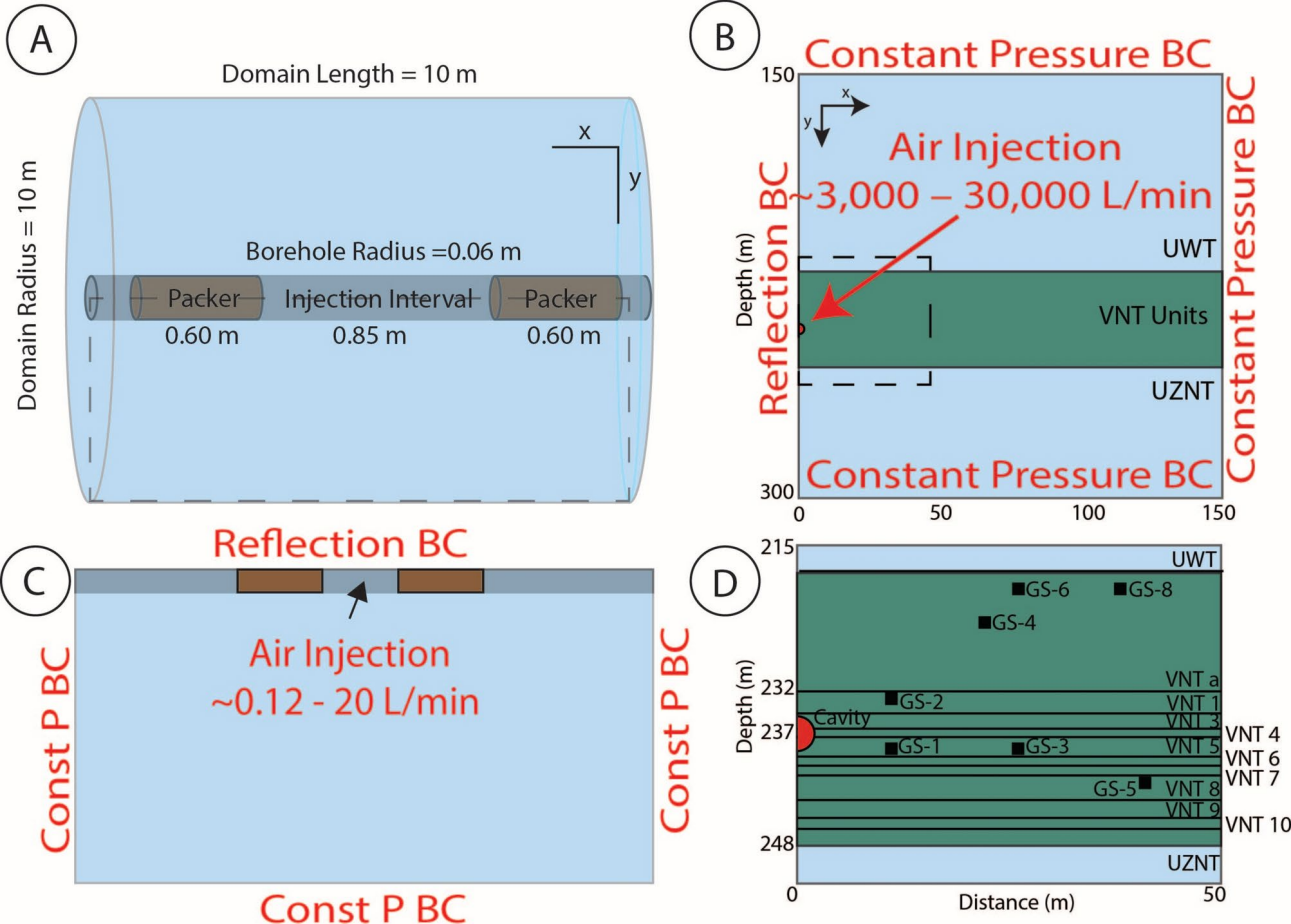
Model domains of the two permeability experiments. (**A**) Dual packer assembly with dashed box showing the model domain (**C**). Boundary conditions (BC) are labeled on the domain edges (Const P = Constant Pressure). Dimensions of packer assemblies differ between tests (Supplementary Figure S1). (**B**) Model domain and boundary conditions of the cavity pressurization test simulation. Dashed box (**B**) shows the VNT units and positions of the GS pressure transducers (**D**). Translation of GS-1–8 preserves the distance from the cavity to the sampler and the host VNT layer.

The packer test domain extends 10 m radially and 10 m in length. The center of the borehole lies along the x = 0 m axis with 0.06 m radius (Fig. 2). Cell size in the x-direction varies from 1 cm within the borehole to Δx = 0.1 m and spacing increases geometrically by a factor of 1.25 radially away from the borehole to a maximum Δx of 2 m. The Δy is constant at 5 cm spacing.

Position, length, and number of the packers in the model are dependent on the packer assembly and change for each set of packer tests (Supplementary Figure S1). The packers are assumed to be impermeable (k = 1E-21 m^2^), and the surrounding rock is homogeneous with properties corresponding to the unit where the test was performed. Hydraulic properties (saturation, porosity, and grain density) are averaged for VNT layers 3–5 from data collected from grab samples (Table 1). Saturation and grain density have not been analyzed experimentally for the other VNT layers so we use the average of the three analyzed VNT layers, 65% saturation and density of 2,260 kg/m^3^. The hydraulic properties of the packers, VNT layers, and borehole void space are reported in Table 1. Simulations were isothermal since we do not expect a large change in temperature at the scale or pumping rates of the packer tests.

**Table 1:** Physical properties and multi-modal van Genutchen parameters^41^ of simulated units/features. Results for in situ water content, thermogravimeteric analysis, and gas and mercury porosimetry on P-Tunnel grab/core samples are in parentheses^11^. Average of saturation and density values from other VNT units (0.65 and 2260, respectively) are used for VNT1, 6–10.

Geologic Unit/Feature	Saturation [-]	Grain density [kg/m^3^]	Porosity [-]	Log_10_(α_1_, α_2_) [Pa^-1^]	m_1_, m_2_ [-]
VNTa	0.65 (—)	2260 (—)	0.36 (—)	4.297, 6.585	0.700, 0.408
VNT1	0.65 (—)	2260 (—)	0.36 (0.36)	4.297, 6.585	0.700, 0.408
VNT3	0.43 (0.4–0.48)	2290 (2280–2300)	0.35 (0.18–0.35)	4.297, 6.585	0.700, 0.408
VNT4	0.65 (0.51–0.92)	2203 (2200–2210)	0.30 (0.20–0.35)	4.297, 6.585	0.700, 0.408
VNT5	0.76 (0.61–0.89)	2316 (2240–2380)	0.37 (0.25–0.42)	4.297, 6.585	0.700, 0.408
VNT6	0.65 (—)	2260 (—)	0.32 (0.32)	4.297, 6.585	0.700, 0.408
VNT7	0.65 (—)	2260 (—)	0.24 (0.24)	4.297, 6.585	0.700, 0.408
VNT8	0.65 (—)	2260 (—)	0.32 (0.32)	4.297, 6.585	0.700, 0.408
VNT9	0.65 (—)	2260 (—)	0.24 (0.24)	4.297, 6.585	0.700, 0.408
VNT10	0.65 (—)	2260 (—)	0.16 (0.16)	4.297, 6.585	0.700, 0.408
Packer^1^	0.001	1000	0.01	-	-
Grout^1^	0.90	2000	0.10	-	-
Borehole^1^	0.001	1000	0.99	-	-
Cavity^2^	0.1	1000	0.21	-	-

^1^Included in packer simulations only.

^2^Included in cavity pressurization simulations only.

Constant pressure (type 2 Neumann) boundary conditions are enforced on the longitudinal boundaries of the domain and a constant air flux (${Q}_{a}$; Eq. 1) boundary condition is used in the injection chamber. A reflection boundary condition is used on the radial boundary. Depending on the packer test, pumping rates ranged between 0.1 to 20 L/min. Boundary effects are not observed to influence the model at pumping rates of 20 L/min (see Supplementary Figure S4).

An extensive description of the methodology of the PEST calibration used in this study is found in the Supplementary Information section. To summarize here, we allow PEST to run the FEHM simulation and adjust the VNT permeability with the assumption that the permeability value is between 1E-12 to 1E-18 m^2^ (initially $k$ = 1E-15 m^2^ or ~ 1 mD). After comparing the simulated versus observed steady-state pressure within the injection interval using the Gauss-Marquardt–Levenberg method^51^, PEST calculates a new permeability for the FEHM simulation. This iterative process continues until PEST minimizes the error between the observed and simulated data, known as the objective function.

### Cavity pressure test model design and assumptions

The cavity pressurization test is simulated using FEHM on a 150 m by 150 m mesh to avoid boundary effects. The element length discretization of the tetrahedral mesh is uniform (Δx = Δy = 1 m) except within the cavity (Δx = Δy = 0.25 m). The mesh is constructed using the LANL gridding tool LaGrit^54^.

The spherical cavity lies within the VNT layers. The positions of the GS-1–8 samplers are translated from 3D space onto 2D space based on their residing VNT layer and distance from the cavity center (Fig. 2). The cavity is assumed to be filled with dry sand that is highly permeable. We allow the permeability to vary from 1E-9 to 1E-12 in the calibration process. The saturation, porosity and density of the VNT layers are assigned using data collected from SNL (Table 1). The timestep size varied from 0.0086 to 650 s. Constant pressure boundary conditions (type 1; Dirichlet) are assumed on the top, bottom, and right lateral edge of the model domain (P = 0.08 MPa; atmospheric pressure at the NNSS). The effects of air-static gradient and the influence of barometric fluctuations at the land surface were not considered since the forcing from the cavity pressurization is much greater (0.15 MPa). The left lateral edge is a no-flux/reflection (type 2; Neumann) boundary condition, about which the model solution is radially symmetric. Pressurization of the cavity is represented by air injected uniformly within the entire cavity volume as a transient specified flux (type 2; Neumann) boundary condition. The air source term is distributed within the cavity volume and varies from 3,000 to 30,000 L/min over the 2.5-h injection time.

Unlike the packer tests, pressures at the gas samplers in the cavity pressurization test never achieved steady-state pressure due to the time-varying injection rate into the cavity. PEST is provided with 500 randomly selected observation points from the pressure data recorded by the GS samplers from the start of sampling through one day. PEST iteratively adjusts the permeability of the FEHM forward models using the Tikhonov regularization method^50^ until the objective function is minimized.

## Results

### Core measurements

Physical and hydrologic characterization results from core measurements are displayed in Tables 1 and 2. The VNT units remain moderately to nearly fully saturated (40–92%). Overall, porosity decreases with depth from VNT-1 to VNT-10. However, the porosity within one unit may vary considerably. Porosity measurements were taken from multiple cores within VNT-3–5 and range in porosity from 18–42%. Core permeability estimates range two orders of magnitude. MICP measurements were performed on samples from VNT-1, -3, -4, -5, -6, -9, -10. The highest core permeability estimate was observed in VNT-6 (2E-13 m^2^). The variability in permeability of the units captures the heterogeneous nature of tuff hydraulic properties at the local scale.

**Table 2:** Estimated and calibrated permeability across three scales of measurements: (1) Core permeability estimate based on capillary intrusion, (2) packer tests (3) cavity pressurization test. A full list of the 106 packer test measurements in supplemental materials (Supplementary Table S2). All boreholes were used in the packer tests with the exception of DA-1.

Borehole ID	Geologic unit	Core permeability (m^2^)^¥^	Packer test (m^2^)			Cavity pressurization (m^2^)*
			Min	Max	Median	
GS-4*,6*,8*	VNT-a	-	3.34E-13	2.33E-12	7.20E-13	9.86E-14
HF-1, GS-2*	VNT-1	7.22E-14	7.73E-14	4.67E-13	7.95E-14	3.84E-13
GI-2^¥^,3^¥^,4^¥^ HF-1	VNT-3	1.03E-14	4.18E-15	8.90E-14	1.09E-14	2.13E-12
GI-2^¥^,3^¥^,4^¥^, AC-1, HF-1	VNT-4	1.38E-15	3.70E-16	1.91E-14	7.09E-15	4.99E-13
GI-4^¥^,5^¥^,6^¥^, AC-1, HF-1, GS-1*,3*	VNT-5	2.07E-15	1.35E-15	1.62E-13	1.39E-14	2.59E-14
GI-6, DA-1¥	VNT-6	2.31E-13	2.69E-16	5.42E-14	3.81E-14	2.42E-13
AC-1, GI-6	VNT-7	-	6.55E-16	3.37E-15	1.98E-15	1.74E-13
AC-1, GI-6, GS-5*	VNT-8	-	6.09E-17	9.11E-15	7.98E-16	5.84E-14
AC-1, DA-1^¥^	VNT-9/10	8.68E-15	6.51E-17	7.00E-16	3.96E-16	3.43E-17
**-**	Cavity	-	-	-	-	1.00E-11

^¥^ Boreholes correspond to laboratory mercury intrusion tests.

*Boreholes correspond to cavity pressurization test.

### Packer test

Calibrated permeability values from packer tests demonstrate the spatial variability of flow properties between and within the VNT units. Table 2 reports the permeability range for each VNT unit. Maximum, minimum and median permeability values are reported to demonstrate the range of permeability in each unit. Multiple packer tests were performed in the AC, HF, and GI boreholes to characterize multiple VNT units with one borehole.

In the shallower units (VNT-a-5), the calibrated permeability fits the data reasonably well. The maximum residual, difference between the observed and simulated pressure, is between 0.039 to 260 Pa. Calibrated permeabilities of VNT-4 and -5 span across two orders of magnitude. These VNT layers were characterized by packer tests in 5–7 boreholes and show spatial heterogeneity.

Below the VNT layers, the tuff has been zeolitized (UZNT) due to historical fluctuations of the water table and geochemical interaction with groundwater^14^. Prothro (2018)^14^ notes that the contact between the VNT and UZNT is a transitional contact, and the VNT units lie within this transitional boundary. The weathering of the tuff has left zeolites (clinoptilolite) and other argillic clays in the pores, reducing the porosity of the UZNT^14^. This permeability-depth relationship is observed in the deeper VNT units, where VNT-7–10 are consistently less permeable and possess higher maximum residual error. Due to the low porosity within VNT-9 and -10, slight variations in flow rates lead to large fluctuations in interval pressure. Within the VNT-9/10 boundary, packer tests in the AC-1 borehole used two different injection rates and saw a 72.5 kPa pressure difference between the two tests. In the calibration process, small adjustments to permeability lead to large non-linear pressure deviations and consequently, higher residual error (See Supplementary Figure S5).

### Cavity pressurization test

**Fig. 3:**
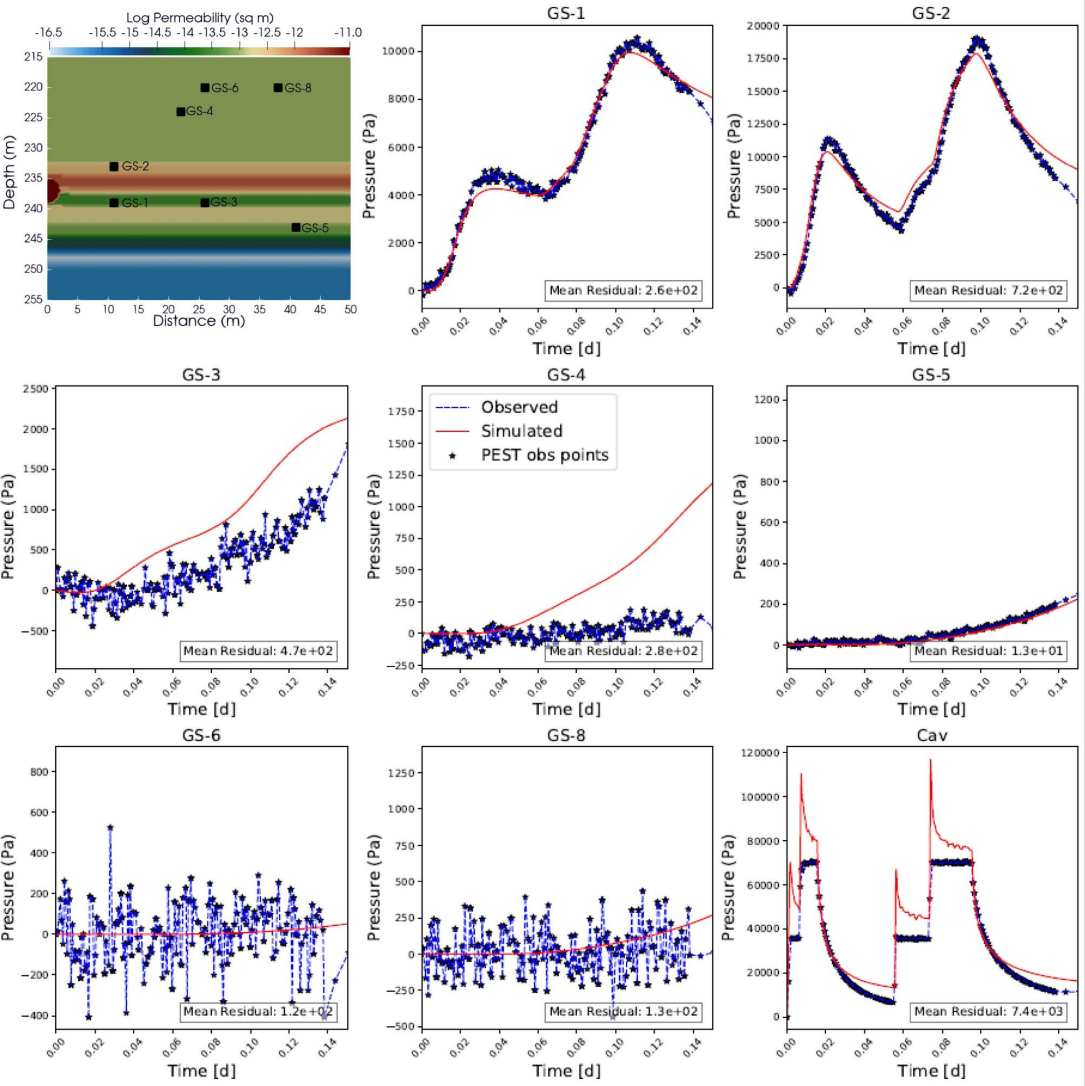
Calibrated permeability from the cavity pressurization test (top left panel; values from Table 2, S2). Four VNT units contain GS samplers: VNT1 (GS-2), VNT5 (GS-1 and 3), VNT 8 (GS-5) and VNTa (GS-4, 6 and 8). Pumping rates into the cavity during the test are shown in Figure S2. Simulated (dashed lines) versus observed (solid lines) pressure signals at GS samplers and cavity during the cavity pressurization test from PEST calibration. The pressure observations used as PEST calibration targets are shown as black stars.

The VNT permeability values determined from the cavity pressurization test PEST calibration were much higher than the packer test calibration results (Table 2). During the experiment, seven pressure transducers (GS-1–8, excluding 7) recorded data within four of the ten VNT units (VNT-a, -1, -5 and -8). GS-7 developed a leak post emplacement and recorded ambient pressure. Figure 3 shows the observed versus simulated pressure signals and mean residual at each sensor used in the cavity pressurization experiment. The PEST calibration results in a geometric mean residual of 281 Pa for all observation groups for the VNT layers.

**Fig. 4:**
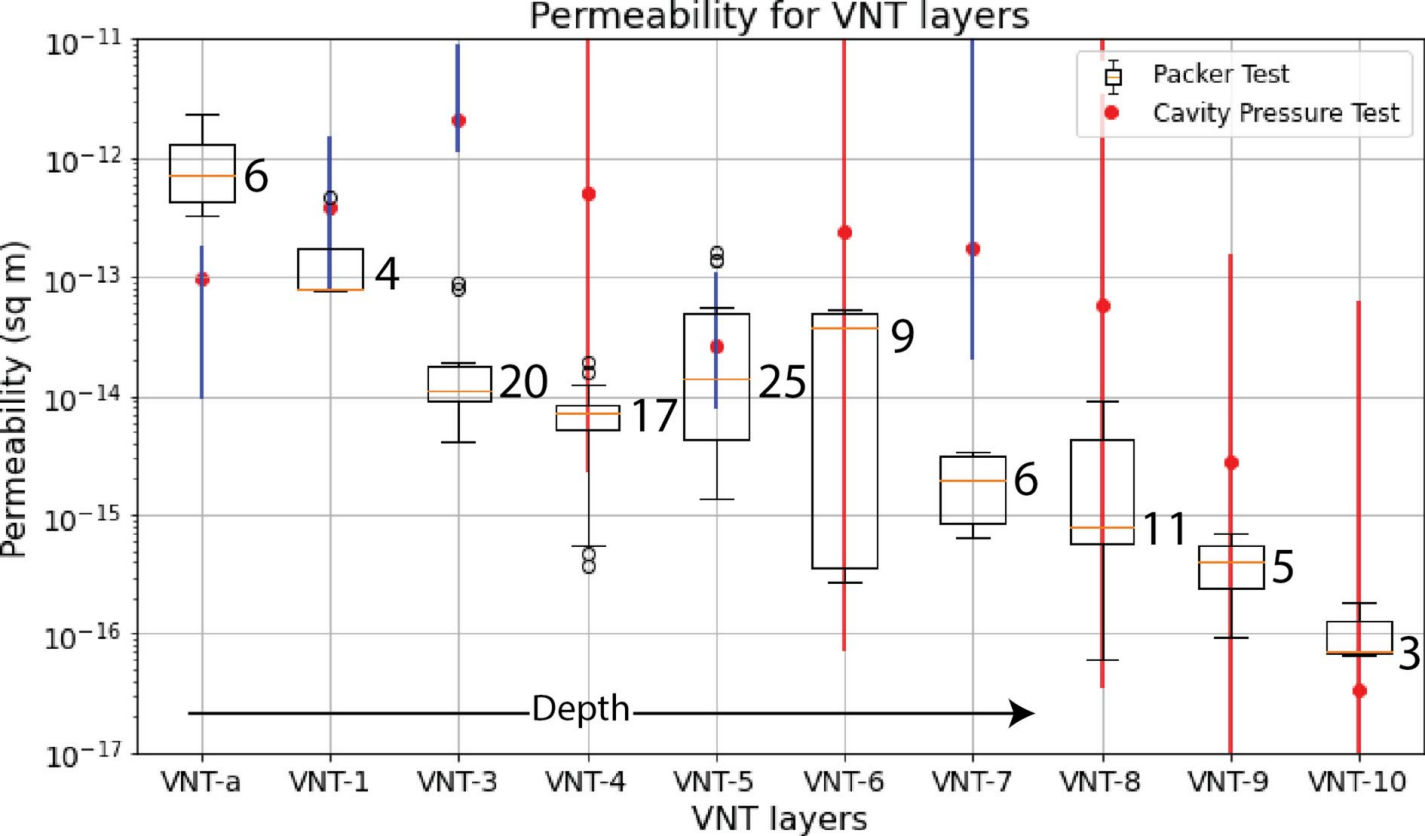
Composite VNT permeability obtained from PEST/FEHM numerical models in this study (116 measurements total). VNT-a is the shallowest unit. Depth increases from VNT-a to VNT-10. The line, whiskers and black open circles of the box plots are the median, 95% confidence interval and outliers of the packer test permeability distribution. Circle and error bars for the cavity pressurization test represent the calibrated permeability and 95% confidence interval, respectively. Error bars that extend beyond the range of PEST permeability estimates are labeled in red, indicating they are relatively insensitive to the calibration process.

Calibrated cavity pressurization tests consistently yield higher permeability data than packer tests in all units except VNT-a and VNT-10 (Fig 4). Red error bars indicate that adjustments to that VNT unit made little difference to the final pressure signal at the GS samplers. Blue error bars show the 95% confidence intervals^52^. The red circles are the values used in the final calibration. Upscaling from the packer test to the cavity pressurization test increases the length scale from 2 m (packer) to 10 – 40 m (cavity), with the potential of incorporating numerous heterogeneous features. Both tests show a permeability-depth relationship (Fig 4). However, contrary to the packer tests, VNT-3 shows the highest permeability (*k* = 2.13E-12 m^2^) instead of VNT-a (2.33E-12 m^2^). High permeability in VNT layers adjacent to the cavity (VNT-3–5) is necessary for the pressure to rapidly bleed off.

## Discussion

### Impact of choice of permeability measurement scale on flow and transport

**Fig. 5:**
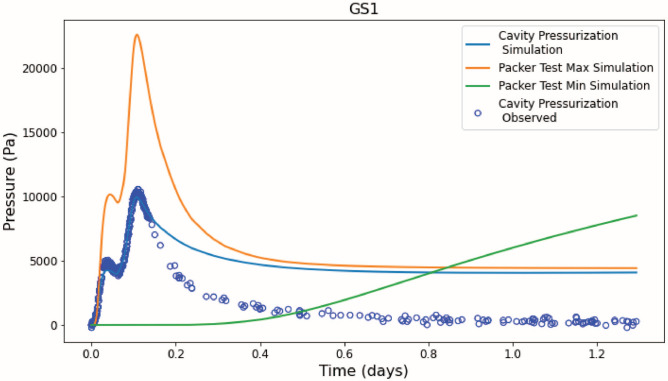
Simulated (lines) and observed (blue dots) pressure signal at GS-1 during the cavity pressurization test with three different permeability scenarios: 1) Inverted permeability from the cavity pressurization test, 2) maximum and 3) minimum inverted permeability from the packer tests.

Scaling effects of hydraulic properties can have a profound impact how representative our models are for subsurface flow and transport. The maximum and minimum permeability derived from the packer inverse models both over- and underestimate the GS-1 pressure signal during the cavity pressurization test (Fig. 5). Except for VNT-a, -1, and -5, permeability calibrated via the cavity pressurization test are orders of magnitude higher than all permeabilities calibrated from the packer tests. To replicate the pressure signals of the GS samplers, the permeability of the VNT units adjacent to the cavity must be high, around one darcy, and capped by lower permeability layers above (VNT-a) and below (VNT-5).

During the cavity pressurization test, the three GS samplers in VNT-a did not observe a measurable change in pressure and were difficult to calibrate. These three pressure transducers fluctuate between + /- 200 Pa with no change after pumping is stopped. No change in pressure signal during the cavity pressurization test indicates either 1) the permeability is too low, preventing the pressure signal from reaching the GS sampler, or 2) VNT-a is too permeable, causing the pressure signal to attenuate before reaching the sampler. The latter is the more likely, given that the packer permeability estimates are around one darcy. Our results suggest that because of the complexity of hydraulic properties and heterogeneous nature of tuff, it is best practice is to calibrate permeability at the scale of interest.

### Limitations of cavity pressurization inversion models

The calibration process for the cavity pressurization test results in permeabilities are non-unique. VNT-a will be slightly higher (1E-13 m^2^ instead of 9E-14 m^2^) if the cavity permeability is decreased from 1E-11 m^2^ to 6E-12 m^2^. However, decreasing cavity permeability will result in higher permeability in all VNT units to reproduce pressure decay around the cavity. Additional errors may be due to the presence of asymmetric fast pathways in the matrix or fractures that cannot be adequately accounted for on an axisymmetric geometry. The relative proximity of samplers to one another is not preserved in 3D (e.g. GS-4 and GS-6 may not be as close as the mesh implies – only their relative distance from the cavity).

Additional uncertainty analyses were conducted to understand the impact of porosity and saturation ranges found in core analyses. The recalibrated permeabilities were within twice the calibrated permeability values when using the average porosity and saturation values. The sensitivity study is described in the Supplementary Information section.

### Insight into permeability scaling: local packer tests versus cavity pressurization tests

When the hydraulic properties of a rock are conceptualized as a continuum, scaling effects can occur by the gradual incorporation of heterogeneities as the scale of the measurement increases. Scaling effects will continue to increase until the scale reaches the representative elementary volume (REV) and the scale effect will reach a maximum.

**Fig. 6:**
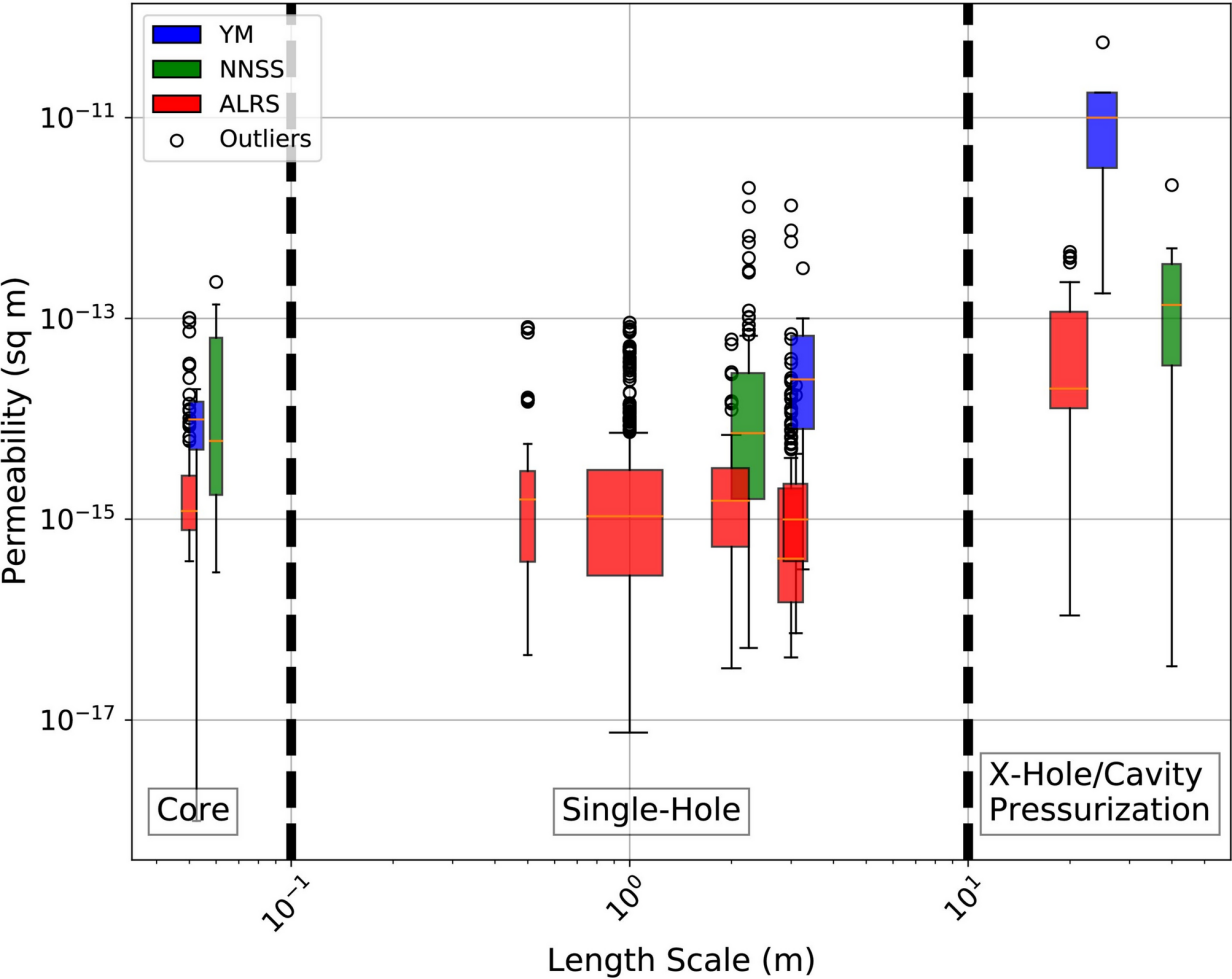
Permeability estimates of volcanic tuffs performed at three scales: 1) laboratory-analyzed core samples, 2) single-hole packer tests (calibrated), and 3) cross-hole pumping tests/cavity pressurization test (calibrated). The orange line, whiskers and black circles of the box plots are the median, 95% confidence interval and outliers of the permeability distribution. The data were obtained from the Apache Leap Research Site, AZ (ALRS, 1309 measurements)^25,56,57^, Yucca Mountain, NV (YM, 25 measurements)^24,55^ and the Nevada National Security Site, NV (NNSS, 116 measurements in this study and 8 measurements in Reference 11). Scale of the measurements are based on the size of the core, pressurized interval in packer tests, and maximum distance between packers in cross-hole and cavity pressurization tests.

Upon further examination of scale-related permeability measurements in volcanic tuff, we begin to see a general trend. Figure 6 shows the permeability of tuffs within this study (NNSS), Yucca Mountain (YM), NV^24,54^, and the ALRS, AZ^25,56,57^, over 1400 + measurements in total. Outliers in the tuff permeability distribution are present at all scales (outside the 95% confidence interval) and are represented by the open circles in the box plot diagram. The outliers are all above the median, represented by the orange line, indicating high permeability. Even with outliers taken into consideration, no statistically significant changes in permeability were observed as the packer interval increased from 0.5 to 20 m in a single well test^29^. The outliers likely represent high permeability features within the volcanic tuff matrix that may be missed without dense sampling. A single individual discrete high permeability conduit from the cavity could be invoked to rapidly drain pressure; however this scenario would not fit the fact that all discrete monitoring points located within a 10’s of meter radial 3-D volume sampled around the injection cavity see rapid pressure changes. Thus, to fit the data at the seven outlying sample locations, permeability within the entire formation must be higher than core or packer measured permeability.

These previous studies reported that both pneumatic and hydrologic cross-well tests consistently yielded permeability measurements higher than single-well packer tests, despite differing textural characteristics (i.e. welding and zeolitization) across each study area, a result that is echoed in our study. At the local scale, regions with higher permeability could connect to form pathways that contribute to an overall increase in bulk permeability across the cross-borehole scale.

The ALRS tuffs are partially welded ash-flow deposits that are heavily fractured^58^, whereas the Crater Flat and Bullfrog tuffs of Yucca Mountain are non-welded and zeolitized^55^. As discussed previously, the VNT units at P-Tunnel are vitric and non-welded, however zeolites are more present with increasing depth. Within volcanic tuff, scaling effects appear relevant at scales greater than 10 m across these three study areas (Fig. 6).

Previous authors have attributed scaling effects from the laboratory scale to the borehole scale to 1) the skin effects that describe the influence of the well on the pressure field^25^, 2) turbulence near sampling ports and injection boreholes^25–28,57^ 3) the dual-continuum porous media structures introduced by fractures^59^. Skin effects have been shown to have little influence on the pressure field during single- and cross-borehole tests^25^. Because the VNT units are structurally weak, fracturing is unlikely and the damage to the periphery of the cavity during construction is minimal^14^. Furthermore, it would be accounted for in the calibration process since the cavity permeability is an adjustable parameter.

Upscaling permeability estimates is fundamental to modeling contamination transport, underground nuclear explosion monitoring and nuclear waste disposition. Understanding the spatial distribution of permeability, both in terms of magnitude and its variability, is crucial for assessing long-term containment strategies and for developing effective monitoring systems in geologically complex environments like volcanic tuff.

## Conclusions

We present inverted permeabilities derived from numerical models that represent the conditions of experiments performed at multiple spatial scales. The numerical models reproduce steady-state pressures in packer tests (1–2 m-scale) and pressure signals observed in cavity pressurization test (10–40 m scale) performed in P-Tunnel from 2021 to 2023.

The tuff permeability clearly shows a depth-dependence as the shallower nonwelded tuff becomes progressively more zeolitic with increasing depth. We observe via inverse analyses of the tuff units (VNT) that derived permeability varies depending on the scale of measurement. Increasing the volume of the rock over which pressure measurements are performed (i.e., cavity pressurization test) results in higher bulk permeability than localized packer tests. The discrepancy between derived permeabilities from packer and cavity pressurization test is due to the sampling of additional heterogeneity as the spatial scale increases. These scaling effects highlight the importance of accurately characterizing the complex geology of P-Tunnel at multiple scales depending on the scale of the problem of interest. For instance, a similar permeability scaling relationship was not observed when comparing bench-scale core measurements to packer tests. For certain applications localized permeability measurements may be sufficient; on the other hand, if one is interested in predicting far-field flow and transport, simply using multiple scales at the core and packer test level will miss a significant degree of heterogeneity.

Improving multi-phase flow and transport simulations in volcanic tuff supports the PE1 experiment process by optimizing the placement of pressure sensors and gas sampling equipment in future experiments in the P-Tunnel. We demonstrate the impact of measurement scale on the resultant pressure wave propagation through the rock by comparing simulations using permeability measurements collected at two distinct scales. In so doing, we find in this simple comparison that even though measurements at each independent scale were correct, measurements closer to the relevant flow processes of interest provide a much better estimate of pressure propagation. Moreover, characterizing how scaling effects influence hydraulic properties is critical in applying our models to new lesser-known environments for nuclear explosion monitoring, environmental remediation, and nuclear waste disposition.

## Supplementary Information

Supplementary Information.

## Data Availability

Due to the nature of the research, the data that support the findings of this study cannot be made available until after Feb 9, 2026, following an embargo on the public release of the data from the sponsoring organization. After this date, data will be made available via public server or upon reasonable request to the corresponding author: Dolan D. Lucero (dolanlucero@lanl.gov).

## References

[1] Myers, S. C., Abbot, G., Alexander, T., Alger, E., Alvarez, A., et al. A Multi-Physics Experiment for Low-Yield Nuclear Explosion Monitoring. Lawrence Livermore National Laboratory Report. LLNL-TR-864107. (2024).

[2] Sun, Y. & Carrigan, C. R. Modeling noble gas transport and detection for the comprehensive nuclear-test-ban treaty. *Pure Appl. Geophys.* **171**, 735–750. 10.1007/s00024-012-0514-4 (2014).

[3] Carrigan, C. R. et al. Delayed signatures of underground nuclear explosions. *Sci. Rep.* **6**, 23032. 10.1038/srep23032 (2016). 26979288 10.1038/srep23032 PMC4793292

[4] Bourret, S. M., Kwicklis, E. M., Harp, D. R., Ortiz, J. P. & Stauffer, P. H. Beyond Barnwell: applying lessons learned from the Barnwell site to other historic underground nuclear tests at Pahute Mesa to understand radioactive gas-seepage observations. *J. Environ. Radioact.* **222**, 106297. 10.1016/j.jenvrad.2020.106297 (2020). 32739734 10.1016/j.jenvrad.2020.106297

[5] Bourret, S. M., Kwicklis, E. M., Miller, T. A. & Stauffer, P. H. Evaluating the importance of barometric pumping for subsurface gas transport near an underground nuclear test site. *Vadose Zone J* **18**(1), 180134. 10.2136/vzj2018.07.0134 (2019).

[6] Jordan, A. B., Stauffer, P. H., Knight, E. E., Rougier, E. & Anderson, D. N. Radionuclide gas transport through nuclear explosion-generated fracture networks. *Sci. Rep.* **5**(1), 18383 (2015). 26676058 10.1038/srep18383 PMC4682097

[7] Jordan, A. B. et al. Uncertainty in prediction of radionuclide gas migration from underground nuclear explosions. *Vadose Zone J.* **13**(10), 1–13 (2014).

[8] Harp, D. R. et al. Immobile pore-water storage enhancement and retardation of gas transport in fractured rock. *Transp. Porous Media* **124**, 369–394 (2018).

[9] Harp, D. R., Ortiz, J. P. & Stauffer, P. H. Identification of dominant gas transport frequencies during barometric pumping of fractured rock. *Sci. Rep.* **9**(1), 9537 (2019). 31267037 10.1038/s41598-019-46023-z PMC6606586

[10] Nilson, R. H., Peterson, E. W., Lie, K. H., Burkhard, N. R. & Hearst, J. R. Atmospheric pumping: A mechanism causing vertical transport of contaminated gases through fractured permeable media. *Journal of Geophysical Research: Solid Earth* **96**(B13), 21933–21948 (1991).

[11] Wilson, J.E., Heath, J.E., Kuhlman, K.L., Xu, G., Bodmer, M.A., Broome, S.T., et al. PE1 Site Characterization: Data Documentation on Geologic and Hydrologic Lab Testing. SAND2024–07526. (2024). 10.2172/2429952

[12] Bodmer, M., Townsend, M., Roberts, B., Wilson, J., Reppart, J., et al. LYNM PE1 Pre-Experiment A Site Characterization Report, SAND2024–07522. (2024). 10.2172/2429935

[13] USDOE. United States nuclear tests, July 1945 through September 1992. DOE/NV-209-Rev 16. Natl. Nucl. Security Admin., Nevada Field Office, Las Vegas. (2015).

[14] Prothro, L. Geologic framework model for the Underground Nuclear Explosions Signatures Experiment, P-Tunnel testbed, Aqueduct Mesa, Nevada National Security Site (NV/DOE/03624–0312). Mission Support and Test Services.LA-UR-22–27846. (2018).

[15] Heikoop, J. M. et al. Isotopic evidence for reduction of anthropogenic hexavalent chromium in Los Alamos National Laboratory groundwater. *Chem Geol* **373**, 1–9. 10.1016/j.chemgeo.2014.02.022 (2014).

[16] Vesselinov, V.V., Katzman, D., Broxton, D., Birdsell,K., Reneau,S., et al. Data and Model-driven Decision Support for Environmental Management of a Chromium Plume at Los Alamos National Laboratory. *Waste Management Symposium* 2013, Session 109, February24–28, 2013, Phoenix, AZ, USA. (2013).

[17] Stauffer, P. H. et al. Evidence for high rates of gas transport in the deep subsurface. *Geophys. Res. Lett.* **46**(7), 3773–3780. 10.1029/2019GL082394 (2019).

[18] Behar, H. R. et al. An investigation of plume response to soil vapor extraction and hypothetical drum failure. *Vadose Zone J.* **18**, 180080. 10.2136/vzj2018.04.0080 (2019).

[19] Stauffer, P. H. et al. Vadose zone transport of tritium and nitrate under ponded water conditions. *Geosciences* **12**, 294. 10.3390/geosciences12080294 (2022).

[20] Bullock, C. A., Chastenet de Gery, M. J., Whicker, J. J., Stanton, J. K., Gruber, C. E., & Valdez, J. M., Sampling and Analysis Plan for TRU Liquid Waste Facility Soil. Los Alamos National Laboratory Report. LA-UR-21–21129. (2021).

[21] Birdsell, K. H., Dai, Z., Stauffer, P. H., & French, S. B. Special Analysis: 2016–002, Analysis of Cover Erosion and Enhanced Infiltration at Pit 25, TA-54 Area G. Los Alamos National Laboratory Report. LA-UR-18–23773. (2019).

[22] Lu, Z. & Stauffer, P. H. On estimating functional average breakthrough curve using time-warping technique and perturbation approach. *Water Resour. Res.* **48**, W05541. 10.1029/2011WR011506 (2012).

[23] Stauffer, P. H., Vrugt, J. A., Turin, H. J., Gable, C. W. & Soll, W. E. Untangling diffusion from advection in unsaturated porous media: Experimental data, modeling, and parameter uncertainty assessment. *Vadose Zone J.* **8**, 510–522. 10.2136/vzj2008.0055 (2009).

[24] Zyvoloski, G. et al. The site-scale saturated-zone flow model for Yucca Mountain: Calibration of different conceptual models and their impact on flow paths. *J. Contam. Hydrol.* **62–63**, 731–750 (2003). 12714319 10.1016/s0169-7722(02)00190-0

[25] Illman, W. A. & Neuman, S. P. Type-curve interpretation of a cross-hole pneumatic test in unsaturated fractured tuff. *Water Resour Res* **37**, 583–604 (2001). 10.1111/j.1745-6584.2001.tb02358.x 11554246

[26] Illman, W. A. & Neuman, S. P. Steady-state analyses of cross-hole pneumatic injection tests in unsaturated fractured tuff. *J Hydrol* **281**, 36–54 (2003).

[27] Vesselinov, V. V., Neuman, S. P. & Illman, W. A. Three-dimensional numerical inversion of pneumatic cross-hole tests in unsaturated fractured tuff: 1. Methodology and borehole effects. *Water Resour. Res.* **37**(12), 3001–3018 (2001).

[28] Vesselinov, V. V., Neuman, S. P. & Illman, W. A. Three dimensional numerical inversion of pneumatic cross-hole tests in unsaturated fractured tuff: 2. Equivalent parameters, high-resolution stochastic imaging and scale effects. *Water Resour. Res.* **37**(12), 3019–3042 (2001).

[29] Neuman, S. P. & Di Federico, V. Multifaceted nature of hydrogeologic scaling and its interpretation. *Rev. Geophys.*10.1029/2003RG000130 (2003).

[30] Chen, G., Illman, W.A., Thompson, D.L., Vesselinov, V.V., & Neuman, S.P. Geostatistical, type curve and inverse analyses of pneumatic injection tests in unsaturated fractured tuffs at the Apache Leap Research Site near Superior, Arizona. In B. Faybishenko et al. (Eds.), Dynamics of Fluids in Fractured Rocks, Geophysical Monograph Series, 122, 73–98. AGU. (2000).

[31] Illman, W. A. Analysis of permeability scaling within single boreholes. *Geophys. Res. Lett.* **31**, L06503 (2004).

[32] Di Federico, V. & Neuman, S. P. Scaling of random fields by means of truncated power variograms and associated spectra. *Water Resour. Res.* **33**(5), 1075–1085 (1997).

[33] Di Federico, V., Neuman, S. P. & Tartakovsky, D. M. Anisotropy, lacunarity, upscaled conductivity and its covariance in multiscale fields with truncated power variograms. *Water Resour. Res.* **35**(10), 2891–2908 (1999).

[34] Sanchez-Vila, X., Carrera, J. & Girardi, J. P. Scale effects in transmissivity. *J. Hydrol.* **183**, 1–22 (1996).

[35] Hyun, Y. et al. Theoretical interpretation of a pronounced permeability scale effect in unsaturated fractured tuff. *Water Resour. Res.* **38**(6), 1082. 10.1029/2001WR000658 (2002).

[36] Tidwell, V. C. & Wilson, J. L. Upscaling experiments conducted on a block of volcanic tuff: Results for a bimodal permeability distribution. *Water Resour. Res.* **35**(11), 3375–3387 (1999).

[37] Bredehoeft, J. D., Neuzil, C. E. & Milly, P. C. D. Regional flow in the Dakota Aquifer: A study of the role of confining layers. *U.S. Geol. Survey Water Supply Paper.* **2237**, 45 (1983).

[38] Neuzil, C. E. How permeable are clays and shales?. *Water Resour. Res.* **30**(2), 145–150 (1994).

[39] Neuzil, C. E. Permeability of clays and shales. *Annu. Rev. Earth Planet. Sci.* **47**, 247–273 (2019).

[40] Galvão, P., Halihan, T. & Hirata, R. Evaluating karst geotechnical risk in the urbanized area of Sete Lagoas, Minas Gerais, Brazil. *Hydrogeol. J.*10.1007/s10040-015-1266-x (2015).

[41] Heath, J. E., Kuhlman, K. L., Broome, S. T., Wilson, J. E. & Malama, B. Heterogeneous multiphase flow properties of volcanic rocks and implications for noble gas transport from underground nuclear explosions. *Vadose Zone J.*10.1002/vzj2.20123 (2021).

[42] Neil, C. W. et al. Gas diffusion through variably-water-saturated zeolitic tuff: Implications for transport following a subsurface nuclear event. *J. Environ. Radioact.* **250**, 106905 (2022). 35598406 10.1016/j.jenvrad.2022.106905

[43] Sawyer, D. A. et al. Episodic Caldera Volcanism in the Miocene southwest Nevada volcanic field: revised stratigraphic framework, 40Ar/39Ar geochronology and implications for magmatism and extension. *Geol. Soc. Am. Bull.* **106**, 1304–1318 (1994).

[44] Cole, J. C., & Cashman, P. H. Structural Relationships of Pre-Tertiary Rocks in the Nevada Test Site Region, Southern Nevada. U.S. Geological Survey Professional Paper 1607. (1999).

[45] Prothro, L. B., Drellack, S. L., Jr., & Mercadante, J. M. A Hydrostratigraphic System for Modeling Groundwater Flow and Radionuclide Migration at the Corrective Action Unit Scale, Nevada Test Site and Surrounding Areas, Clark, Lincoln, and Nye Counties, Nevada. DOE/NV/25946--630, Las Vegas, NV. (2009).

[46] Swanson, B. F. A simple correlation between permeabilities and mercury capillary pressures. *J. Petrol. Technol.* **33**, 2498–2504 (1981).

[47] Fritz, B.G., Peterson, J.A., Munley, W.O., & Boukhalfa, H. Description of the Gas Sampling and Circulation System for the LYNM PE1-A Experiment.PNNL-36268.Pacific Northwest National Laboratory, Richland, WA. (2024).

[48] Zyvoloski, G.A., Robinson, B.A., Dash, Z.V., Kelkar, S., Viswanathan, H.S., Pawar, R.J., ... & Stauffer, P.H. Software Users Manual (UM) for the FEHM Application Version 3.1 - 3.X, Rev. 2, LANL Report LA-UR-12–24493. (2015).

[49] FEHM. Retrieved from https://fehm.lanl.gov/, accessed December 21, 2023. (2023).

[50] Doherty, J. E. PEST: Model-Independent Parameter Estimation and Uncertainty Analysis—User Manual Part I. Watermark, Brisbane, Australia. (2021).

[51] Marquardt, D. W. An algorithm for least-squares estimation of nonlinear parameters. *J. SIAM* **11**, 431–441 (1963).

[52] Doherty, J. E. Calibration and uncertainty analysis for complex environmental models. *Watermark Numerical Computing*, Brisbane, Australia. 227pp. ISBN: 978–0–9943786–0–6 (2015).

[53] Pebesma, E. J. & C G.,. Wesseling GSTAT: a program for geostatistical modelling, prediction and simulation. *Comput. Geosci.* **24**(1), 17–31 (1998).

[54] Los Alamos Grid Toolbox (LaGriT). Los Alamos National Laboratory. Retrieved from http://lagrit.lanl.gov (2019).

[55] Geldon, A.L. Results and Interpretation of Preliminary Aquifer Tests in Boreholes UE-25c #1, UE-25c #2, and UE-25c #3, Yucca Mountain, Nye County, Nevada. Water-Resources Investigations Report 94–4177. US Geological Survey, Denver, CO. (1996).

[56] Rasmussen, T. C., D. D. Evans, P. J. Sheets, & J. H. Blanford, Unsaturated fractured rock characterization methods and data sets at the Apache Leap Tuff Site, Rep. NUREG/CR-5596, U.S. Nucl. Regul. Comm., Washington, D. C., (1990).

[57] Guzman, A., Neuman, S. P., Lohrstorfer, C., & Bassett, R. L. Validation studies for assessing flow and transport through unsaturated fractured rocks. In R. L. Bassett et al. (Eds.), Rep. NUREG/CR-6203, chap. 4. U.S. Nucl. Regul. Comm., Washington, D.C. (1994).

[58] Peterson, D.W. Dichotic Ash-Flow Sheet near Superior and Globe, Arizona, Doctoral Dissertation, Dept. of Geology, Stanford Univ., 130 pp, maps. (1961)

[59] Rovey, C. W. II. Digital simulation of the scale effect in hydraulic conductivity. *J. Hydrol.* **6**, 216–225 (1998).

