# Supplementary material for: Permeability scaling relationships of volcanic tuff from core to field scale measurements
Source: Sci Rep. 2025 Apr 15;15:12938. doi: 10.1038/s41598-025-96835-5 (PMC12000278; doi:10.1038/s41598-025-96835-5)
Supplement: Supplementary file 1 — Supplementary Information. [file 41598_2025_96835_MOESM1_ESM.docx]

# Permeability Scaling Relationships of Volcanic Tuff from Core to Field Scale Measurements

Dolan D. Lucero^1^, S. Michelle Bourret^1^, John P. Ortiz^1^, Bradley G. Fritz^2^, Miles A. Bodmer^3^, Jason E. Heath^3^, Kristopher L. Kuhlman^3^, Hakim Boukhalfa^1^, Shawn Otto^1^, Souheil Ezzedine^4^, Barry L. Roberts^3^, R. Charles Choens^3^, Mark A. Person^5^, Philip H. Stauffer^1^, and PE1 Experimental Team

1. Los Alamos National Laboratory
2. Pacific Northwest National Laboratory
3. Sandia National Laboratories
4. Lawrence Livermore National Laboratory
5. New Mexico Institute of Mining and Technology

# Supplementary Information

## Packer Test Inverse Models: Manual Regularization

Inverse models use observation data to find solutions for parameter data. Field-scale studies of subsurface hydrogeology are parameter-rich, with a broad range of intra-unit heterogeneity in porous media flow properties. Conversely, observation data can be relatively poor due to the inability to sample data at the scale at which spatial heterogeneities exist. Moreover, estimated parameters are non-unique, requiring uncertainty analysis and expert knowledge to ensure correctness despite measurement error in the observation dataset. Uncertainty analysis and inverse models have the unique capability to elucidate the often non-linear relationship between the parameters and observation points.

A linear, well-posed inverse model can be described as:

$$\boldsymbol{h}=\boldsymbol{Zk-\varepsilon}(S1)$$

where $\boldsymbol{h}$ is the observation data vector, $\boldsymbol{Z}$ is the model coefficient matrix acting on the parameter vector, $\boldsymbol{k}$, and $\boldsymbol{\varepsilon}$ is the measurement error in the observation data. Solutions to $\boldsymbol{k}$ will be non-unique if the matrix $\boldsymbol{Z}$ possesses a null-space (where $\boldsymbol{Z}$ = 0) which is often the case in environmental problems. Consequently, we must use regularization to find uniqueness by reducing the error between model solutions and the calibration dataset. PEST allows the user to estimate parameters using manual or mathematical regularization^1^.

Manual regularization requires the user to determine the number of estimable parameters, lump the parameters, or freeze calibration data at a constant if the values are insensitive or unrealistic. Further, manual regularization requires adjustment of the weighting of each observation point which should be based on the measurement noise. The cost of this flexibility is the potential to introduce user error that could propagate throughout the inversion process.

Within the context of the packer test inverse models, $\boldsymbol{h}$ is the steady-state pressure data in the packer (Table S2), $\boldsymbol{Z}$ is the numerical model (described in sections 2.2 and 2.3) with VNT permeability of $\boldsymbol{k}$**.** Each of the 105 packer tests has a separate FEHM model and is analyzed through manual regularization using PEST to understand the permeability range of each VNT layer.

If the $\boldsymbol{Z}$ matrix is square or overdetermined (# of observations ≥ # of parameters), the estimated parameter set of VNT permeability, $\boldsymbol{k}$**,** may be calculated by:

$$\underline{\boldsymbol{k}}\boldsymbol{=}\left( \boldsymbol{Z}^{\boldsymbol{t}}\boldsymbol{QZ} \right)^{\boldsymbol{-1}}\left( \boldsymbol{Z}^{\boldsymbol{t}}\boldsymbol{QZ} \right)\boldsymbol{h}(S2)$$

where $\boldsymbol{Q}$ is the weighting matrix for each observation and parameter. The selection of the weighting matrix values should be dependent on the measurement noise ($\boldsymbol{\varepsilon}$). If the $\boldsymbol{Z}$ matrix is underdetermined (# of observations ≤ # of parameters), single-value decomposition must be implemented to ensure numerical stability. Single-value decomposition separates $\boldsymbol{Z}$ into workable components to ensure invertibility and will be discussed later in this section. $\underline{\boldsymbol{k}}$ should be calculated so that it minimizes the objective function:

$$\boldsymbol{\Phi=}\mathbf{r}^{\mathbf{t}}\mathbf{Qr =}\left( \mathbf{h - Z}\underline{\mathbf{k}} \right)^{\mathbf{t}}\mathbf{Q}\left( \mathbf{h - Z}\underline{\mathbf{k}} \right) (S3)$$

where $\mathbf{r}$ is the residual, the difference between the simulation output ($\mathbf{Z}\underline{\mathbf{k}}$) and observation data. An iterative approach is taken to reduce the objective function, where PEST experiments with many $\underline{\mathbf{k}}$ values. Ideally, the model will be calibrated when the objective function falls below the measurement noise ($\boldsymbol{\varepsilon}$). However, this is difficult to accomplish, especially if the model is non-linear.

Regularization demands model linearity to calculate parameters, however this is rarely the case in environmental problems. Non-linear models must undergo Taylor Series expansion to approach linearity. Expansion of equation S1 yields:

$$\boldsymbol{h}=\boldsymbol{h}_{\boldsymbol{o}}\boldsymbol{+J\partial k+}other terms (S4)$$

here, the model $\boldsymbol{Z}$ matrix is replaced by $\boldsymbol{J}$ or the Jacobian matrix^2^, $\boldsymbol{h}_{\boldsymbol{o}}$ is the model output of the previous iteration ($\boldsymbol{h}_{\boldsymbol{o}}\boldsymbol{=Z[}\boldsymbol{k}_{\boldsymbol{o}}\boldsymbol{]}$), $\boldsymbol{k}_{\boldsymbol{o}}$ is the previous set of parameters, and $\boldsymbol{\partial k}\boldsymbol{=k-}\boldsymbol{k}_{\boldsymbol{o}}$. The Jacobian matrix, $\boldsymbol{J}$, is an evaluation of model behavior over parameter space.

$$\boldsymbol{J=}\left[ \begin{matrix} \frac{\boldsymbol{\partial}\boldsymbol{Z}_{\boldsymbol{1}}}{\boldsymbol{\partial}\boldsymbol{k}_{\boldsymbol{1}}} & \frac{\boldsymbol{\partial}\boldsymbol{Z}_{\boldsymbol{1}}}{\boldsymbol{\partial}\boldsymbol{k}_{\boldsymbol{2}}} & \frac{\boldsymbol{\partial}\boldsymbol{Z}_{\boldsymbol{1}}}{\boldsymbol{\partial}\boldsymbol{k}_{\boldsymbol{m}}} \\ \frac{\boldsymbol{\partial}\boldsymbol{Z}_{\boldsymbol{2}}}{\boldsymbol{\partial}\boldsymbol{k}_{\boldsymbol{1}}} & \boldsymbol{\ddots} & \boldsymbol{\vdots} \\ \frac{\boldsymbol{\partial}\boldsymbol{Z}_{\boldsymbol{n}}}{\boldsymbol{\partial}\boldsymbol{k}_{\boldsymbol{1}}} & \boldsymbol{\cdots} & \frac{\boldsymbol{\partial}\boldsymbol{Z}_{\boldsymbol{n}}}{\boldsymbol{\partial}\boldsymbol{k}_{\boldsymbol{m}}} \end{matrix} \right] (S5)$$

where $\boldsymbol{m}$ number of parameters and $\boldsymbol{n}$ number of observation points exist. Truncating equation 9 and solving for $\boldsymbol{k-}\boldsymbol{k}_{\boldsymbol{o}}$ we get the parameter improvement equation:

$$\boldsymbol{k-}\boldsymbol{k}_{\boldsymbol{o}}\boldsymbol{=}{\boldsymbol{(J}^{\boldsymbol{t}}\boldsymbol{QJ)}}^{\boldsymbol{-1}}\boldsymbol{J}^{\boldsymbol{t}}\boldsymbol{Qr}(S6)$$

$\boldsymbol{J}$ is calculated each time using the previous parameter set, $\boldsymbol{k}_{\boldsymbol{o}}$. The Marquardt Lambda ($\boldsymbol{\lambda}$) enhances terms in $\boldsymbol{(J}^{\boldsymbol{t}}\boldsymbol{QJ)}$ to ensure that the matrix is diagonally dominant. Equation S6 becomes:

$$\boldsymbol{k-}\boldsymbol{k}_{\boldsymbol{o}}\boldsymbol{=}{\boldsymbol{(J}^{\boldsymbol{t}}\boldsymbol{QJ +}\boldsymbol{\lambda I}\boldsymbol{)}}^{\boldsymbol{-1}}\boldsymbol{J}^{\boldsymbol{t}}\boldsymbol{Qr}(S7)$$

Over successive iterations, PEST calculates multiple parameter upgrades ($\boldsymbol{k-}\boldsymbol{k}_{\boldsymbol{o}}$) using different Marquardt Lambda values to lower the objective function, a process known as the Gauss-Marquardt-Levenberg method^3^. Initially, large values of $\boldsymbol{\lambda}$ lower the objective function then lower values must be used as it approaches the minima or it may overshoot. Manual regularization continues this process until the objective function can no longer be minimized. The VNT inverse models use an initial Marquardt Lambda of 10 that is reduced by a multiplier as it approaches the minimum value of $\boldsymbol{\Phi}$.

## Cavity Pressurization Inverse Models: Mathematical Regularization

Mathematical regularization has the advantage of automatically lumping parameters and adjusting the weighting of observation points. Best practices of mathematical regularization call for the combined utilization of Tikhonov Regularization and Singular Value Decomposition^2^.

Tikhonov Regularization supplements the observation dataset with *expert knowledge* of the input parameters. *Expert knowledge* can range from initial conditions to non-linear relationships between parameters. With the addition of *expert knowledge* to a linear inverse model, equation S1 becomes:

$$\left[ \begin{matrix} \boldsymbol{h} \\ \boldsymbol{w} \end{matrix} \right]\boldsymbol{=}\left[ \begin{matrix} \boldsymbol{Z} \\ \boldsymbol{Z}_{\boldsymbol{w}} \end{matrix} \right]\boldsymbol{k+}\left[ \begin{matrix} \boldsymbol{\varepsilon} \\ \boldsymbol{\omega} \end{matrix} \right] (S8)$$

where $\boldsymbol{w}$ is the regularization observation dataset, $\boldsymbol{Z}_{\boldsymbol{w}}$ is the regularization model, and $\boldsymbol{\omega}$ is the noise associated with the regularization data. $\boldsymbol{\omega}$ can also be considered a measure of the strength/confidence in the expert knowledge. In this context, the *expert knowledge* dataset we provide to the inverse model is cavity pressure ($\boldsymbol{w}$) and the regularization model is the averaged calibrated VNT permeability values from the packer tests ($\boldsymbol{Z}_{\boldsymbol{w}}$). Matching cavity pressure is crucial but secondary to reproducing the GS sampler pressure signal is ($\boldsymbol{h}$).

In Tikhonov Regularization, the solution to $\underline{\mathbf{k}}$ becomes:

$$\underline{\boldsymbol{k}}\boldsymbol{=}\left( \boldsymbol{Z}^{\boldsymbol{t}}\boldsymbol{QZ+}\boldsymbol{\mu}^{\boldsymbol{2}}\mathbf{Z}_{\mathbf{w}}^{\mathbf{t}}\boldsymbol{Q}_{\boldsymbol{r}}\boldsymbol{Z}_{\boldsymbol{w}} \right)^{\boldsymbol{-1}}\left( \boldsymbol{Z}^{\boldsymbol{t}}\boldsymbol{Qh+}\boldsymbol{\mu}^{\boldsymbol{2}}\mathbf{Z}_{\mathbf{w}}^{\mathbf{t}}\boldsymbol{Q}_{\boldsymbol{r}}\boldsymbol{w} \right) (S9)$$

where $\boldsymbol{\mu}^{\boldsymbol{2}}$ is the multiplier of the regularization weight $\boldsymbol{Q}_{\boldsymbol{r}}$ since its diagonal dominance is not guaranteed. Thus, from equation S8, two objective functions are obtained:

$$\boldsymbol{\Phi}_{\mathbf{m}}\mathbf{=}\left( \mathbf{h - Z}\underline{\mathbf{k}} \right)^{\mathbf{t}}\mathbf{Q}\left( \mathbf{h - Z}\underline{\mathbf{k}} \right) \left( S10a \right)$$

$$\boldsymbol{\Phi}_{\mathbf{r}}\mathbf{=}\left( \mathbf{w -}\mathbf{Z}_{\mathbf{w}}\underline{\mathbf{k}} \right)^{\mathbf{t}}\mathbf{Q}_{\mathbf{r}}\left( \mathbf{w -}\mathbf{Z}_{\mathbf{w}}\underline{\mathbf{k}} \right) (S10b)$$

where $\boldsymbol{\Phi}_{\mathbf{m}}$ is the measurement objective function, identical to equation S3, and $\boldsymbol{\Phi}_{\mathbf{r}}$ is the residual objective function. The total observation function is given by:

$$\boldsymbol{\Phi}_{\mathbf{t}}\boldsymbol{=}\boldsymbol{\Phi}_{\mathbf{m}}\boldsymbol{+}{\boldsymbol{\mu}^{\boldsymbol{2}}\boldsymbol{\Phi}}_{\mathbf{r}} (S11)$$

Rather than finding the multiplier $\boldsymbol{\mu}^{\boldsymbol{2}}$ for the residual objective function, PEST allows the user to assign a $\boldsymbol{\Phi}_{\mathbf{m}}^{\mathbf{t}}$. Once $\boldsymbol{\Phi}_{\mathbf{m}}\boldsymbol{=}\boldsymbol{\Phi}_{\mathbf{m}}^{\mathbf{t}}$, PEST will minimize $\boldsymbol{\Phi}_{\mathbf{r}}$. $\boldsymbol{\Phi}_{\mathbf{m}}^{\mathbf{t}}$ is entirely subjective and its magnitude is unclear until it is calculated in the calibration process. If the target is set too low, overfitting may occur. Here, an initial PEST run evaluated the magnitude of $\boldsymbol{\Phi}_{\mathbf{m}}$ by setting the target to an extremely low value ($\boldsymbol{\Phi}_{\mathbf{m}}^{\mathbf{t}}$ to 1*10^-10^ MPa) and running the simulation until $\boldsymbol{\Phi}_{\mathbf{m}}$ was lowered a few times. The $\boldsymbol{\Phi}_{\mathbf{m}}^{\mathbf{t}}$ was then set 5-10 % lower than the objective function ($\boldsymbol{\Phi}_{\mathbf{m}}^{\mathbf{t}}$ = 1.2E-5 MPa).

If the model is non-linear, equation S9 becomes:

$$\underline{\boldsymbol{k}}\boldsymbol{-}\boldsymbol{k}_{\boldsymbol{o}}\boldsymbol{=}\left( \boldsymbol{J}^{\boldsymbol{t}}\boldsymbol{QJ+}\boldsymbol{\mu}^{\boldsymbol{2}}\mathbf{J}_{\mathbf{w}}^{\mathbf{t}}\boldsymbol{Q}_{\boldsymbol{r}}\boldsymbol{J}_{\boldsymbol{w}}\boldsymbol{+\lambda I} \right)^{\boldsymbol{-1}}\left( \boldsymbol{J}^{\boldsymbol{t}}\boldsymbol{Qh+}\boldsymbol{\mu}^{\boldsymbol{2}}\mathbf{J}_{\mathbf{w}}^{\mathbf{t}}\boldsymbol{Q}_{\boldsymbol{r}}\boldsymbol{w} \right) (S12)$$

If the model is well-posed, the Gauss-Marquardt-Levenberg method can be implemented. If not, Singular Value Decomposition is required and should be used on Equation S8. SVD reduces parameters to only those that are sensitive to the calibration dataset ($\mathbf{h}$). It does so by breaking $\boldsymbol{Z}$ into three matrices.

$$\boldsymbol{Z=US}\boldsymbol{V}^{\boldsymbol{t}} (S13)$$

where $\boldsymbol{U}$ is known as the output space of $\boldsymbol{Z}$, a square, orthonormal matrix with $n$ rows, $V$ is a transposed orthonormal matrix with $m$ rows, known as the input space of $\boldsymbol{Z}$, and $\boldsymbol{S}$ is a matrix with only diagonal elements that are greater than or equal to 0. $n$ and $m$ are the number of observations or parameters, respectively. Values of $\boldsymbol{S}$ are arranged from highest to lowest until $n$ rows or until diagonal values become zero. $\boldsymbol{S}$ may then be partitioned into,

$$\boldsymbol{Z=}\boldsymbol{U}_{\boldsymbol{1}}\boldsymbol{S}_{\boldsymbol{1}}\boldsymbol{V}_{\boldsymbol{1}}^{\boldsymbol{t}}\boldsymbol{+}\boldsymbol{U}_{\boldsymbol{2}}\boldsymbol{S}_{\boldsymbol{2}}\boldsymbol{V}_{\boldsymbol{2}}^{\boldsymbol{t}}\boldsymbol{+\ldots=}\boldsymbol{U}_{\boldsymbol{1}}\boldsymbol{S}_{\boldsymbol{1}}\boldsymbol{V}_{\boldsymbol{1}}^{\boldsymbol{t}} (S14)$$

where $\boldsymbol{S}_{\boldsymbol{2}}\boldsymbol{S}_{\boldsymbol{3}}\boldsymbol{\ldots}\boldsymbol{S}_{\boldsymbol{n}}$ are matrices of 0. The solution to the inverse model can then be calculated by equation S13 is substituted into S8, the two equations become:

$$\underline{\boldsymbol{k}}\boldsymbol{=}\boldsymbol{V}_{\boldsymbol{1}}{\boldsymbol{S}^{\boldsymbol{-}}}^{\boldsymbol{1}}\boldsymbol{U}^{\boldsymbol{t}}\boldsymbol{h+}\boldsymbol{V}_{\boldsymbol{1}}{\boldsymbol{S}^{\boldsymbol{-}}}^{\boldsymbol{1}}\boldsymbol{U}^{\boldsymbol{t}}\boldsymbol{\varepsilon} (S15a)$$

$$\underline{\boldsymbol{k}}\boldsymbol{=}\boldsymbol{V}_{\boldsymbol{1}}{\boldsymbol{S}^{\boldsymbol{-}}}^{\boldsymbol{1}}\boldsymbol{U}^{\boldsymbol{t}}\boldsymbol{w+}\boldsymbol{V}_{\boldsymbol{1}}{\boldsymbol{S}^{\boldsymbol{-}}}^{\boldsymbol{1}}\boldsymbol{U}^{\boldsymbol{t}}\boldsymbol{\omega}(S15b)$$

where $\underline{\boldsymbol{k}}$ is related to the true $\boldsymbol{k}$ by

$$\underline{\boldsymbol{k}}\boldsymbol{=}\boldsymbol{V}_{\boldsymbol{1}}\boldsymbol{V}_{\boldsymbol{1}}^{\boldsymbol{t}}\boldsymbol{k (}S16\boldsymbol{)}$$

$\boldsymbol{V}_{\boldsymbol{1}}\boldsymbol{V}_{\boldsymbol{1}}^{\boldsymbol{t}}$ can be thought of as a resolution matrix that orthogonally projects $\boldsymbol{k}$ onto the solution space as $\underline{\boldsymbol{k}}$. This solution minimizes the null space and produces the $\underline{\boldsymbol{k}}$with the lowest error variance. Once this is calculated, the calibration is considered complete.

## Uncertainty Quantification of Hydraulic Properties

Sensitivity analyses of saturation (S) and porosity (*ɸ*) were performed to understand the uncertainty derived from the hydraulic properties found in cores from P-Tunnel. Porosity and saturation exhibit the largest range of values (Porosity: 16 - 42%; Saturation: 40 - 90%). Using the range of porosity and saturation values, PEST calibrated permeability of packer test numerical models to match the observed steady state pressure. In VNT-5, calibrated permeability varied from 1.000E-15 – 1.698E-15 m^2^ (Figure S3). The recalibrated permeabilities were within twice the calibrated permeability values when using the average porosity and saturation values.

## Boundary Effect Analysis: Packer Simulation

For the packer simulations, FEHM solved the flow and transport equations on a radially symmetric mesh 10 m by 10 m (Figure 1). Constant pressure boundary conditions were placed on the lateral and radial edges of the domain. Flow rates into the injection interval ranged from 0.1 to 20 L/min. To test if the simulations were influenced by edge effects of the domain, we tested packer simulations on 30 m by 30 m mesh. Simulations were tested in the VNT-5 unit with the highest flow rate 20 L/min and boundary effects were determined to be minimal. At 10 m in the larger mesh, excess pressure reaches 40 Pa or 0.005 psi (Figure S4). The assumption of constant atmospheric pressure at the boundary within the original mesh was deemed acceptable, as the pressures at the boundary remained very close to atmospheric even at the highest flow rate.

## Analytical Solutions

## Intrinsic Permeability Calculation from Packer Data

Packer pressure data can be used to solve intrinsic permeability analytically after steady-state pressure is achieved^4^. Several assumptions must be upheld to use the analytical solution 1) the porous medium surrounding the borehole must be homogeneous and isotropic 2) flow is assumed to be laminar, governed by Darcy’s Law, and ellipsoidal within the packer test section.

$$K=\left( \frac{Q_{WF}}{\gamma_{b}} \right)\frac{\mu_{a}\ln\left[ \frac{l}{2r_{o}}+\sqrt{1+\left( \frac{l}{2r_{o}} \right)^{2}} \right]}{\pi l}\left( \frac{P_{b}}{P_{b}^{2}-P_{a}^{2}} \right) (S17)$$

where $K$ is the intrinsic permeability, $Q_{WF}$ is the weighted flow rate, $\gamma_{b}$ is the unit weight of air, $\mu_{a}$ is the viscosity of air, $l$ is the length of the test section, $r_{o}$ is the radius of the borehole, $P_{b}$ is the absolute pressure (gage pressure + atmospheric pressure, $P_{a}$). The borehole properties and interval pressure in equation S17 are obtained from the packer tests. The $\gamma_{b}$ is a function of temperature and pressure and is calculated by:

$$\gamma_{b}=\left( \frac{P_{b}}{R_{b}T_{b}} \right) (S18)$$

where $T_{b}$ is temperature and $R_{b}$ is the gas constant for air (Zeigler; 1976) [4].

#### Comparison to Analytical Solution

Intrinsic permeability is calculated analytically for the August-November 2022 packer tests using equation S17. The intrinsic permeability derived from the numerical and analytical model plot closely along the 1:1 line (Figure S6). Error between analytical and numerical derived permeability ranges from 2-13% (Table S3).

Within unsaturated systems, degree of saturation is an important factor in controlling relative gas permeability. As a pore network becomes saturated, the relative gas permeability decreases due to pores being filled with liquid, thus limiting the pore volume available for gas flow. The effect of saturation can be evaluated from the numerical simulations but is not able to be represented by the analytical solution. However, relative gas permeability in the VNT layers changes very little with saturation (Figure 3), which may explain the high degree of agreement between the permeability values between the analytical and numerical solutions. Saturation in VNT layers varies from 43% in VNT-3 to 78% in VNT-5 and the relative gas permeability changes only by 0.11. Numerically-derived permeability is highly dependent on the relative permeability function. Since the relative gas permeability is close to 1.0 for the range of saturation observed in the VNT layers, the PEST-calibrated permeability closely resembles the analytically-calculated intrinsic permeability. Varying the saturation from 45-80% changes the relative permeability from 0.99 to 0.88.

## The Relative Permeability Function

Modeling of multiphase transport in unsaturated porous media is complex. Flow is not only driven by vapor pressure but also by capillary pressure. The magnitude of capillary suction, or the ability for water to retain water in pores, is dependent on saturation and the pore size. The VNT layers were found to have two distinct pore subsystems, 1) matrix and 2) post-/pre-existing microfractures^5^. These subsystems have a direct effect on capillary pressure, making it difficult to fit a unimodal curve below 40% saturation (Figure S8). Heath et al. (2021)^5^ instead fit a multimodal van Genutchen model (Table S4) to the mercury intrusion and direct air-water measurements data performed on P-Tunnel tuffs to capture the non-linearity. Here, we use the multimodal relative permeability function from Heath et al. (2021)^5^ to simulate multiphase flow in the VNT unit of the cavity pressure and packer tests.

The generalized multimodal van Genutchen model takes the following form^6^:

$$S_{e}=\sum_{i=0}^{N_{p}-1} w_{i}S_{i} (S19)$$

where $S_{e}$ is the effective relative liquid saturation, $N_{p}$ is the total number of pore subsystems, $S_{i}$ is the relative saturation of the subsystem $i$, and $w_{i}$ is the weighting prescribed to the pore subsystem ($\sum w_{i}$ = 1). Relative saturation $S_{i}$ is defined by:

$$S_{i}=\left[ 1+\left( \frac{P_{c}}{\alpha_{i}} \right)^{\frac{1}{1-m_{i}}} \right]^{-m_{i}} (S20)$$

where $P_{c}$ is the capillary pressure and $\alpha$ and $m$ are van Genutchen fitting parameters. For unimodal systems, $i$ = 1. The relative permeability ($k_{r}$) of each phase can then be calculated:

$$k_{r}=S_{e}^{\frac{1}{2}} \left[ \sum_{i} \frac{w_{i}}{\alpha_{i}}\frac{\left[ 1-\left( 1-S_{e}^{\frac{1}{m_{i}}} \right)^{m_{i}} \right]}{\sum_{i} \frac{w_{i}}{\alpha_{i}}} \right]^{2} (S21)$$

The relative permeability curve fit to the P-tunnel tuff mercury intrusion capillary pressure (MICP) data is displayed in figure S7.

## References

[1] Doherty, J. E. (2021). PEST: Model-Independent Parameter Estimation and Uncertainty Analysis—User Manual Part I. Watermark, Brisbane, Australia.

[2] Doherty, J. E. (2015). Calibration and uncertainty analysis for complex environmental models. Watermark Numerical Computing, Brisbane, Australia. 227pp. ISBN: 978-0-9943786-0-6

[3] Marquardt, D. W. (1963) An algorithm for least-squares estimation of nonlinear parameters, J. SIAM, 11, 431-441

[4] Zeigler, T.W. (1976). Determination of rock mass permeability. Technical report S-76-2. Chief of Engineers, U.S. Army office, Washington, DC.

[5] Heath, J. E., Kuhlman, K. L., Broome, S. T., Wilson, J. E., & Malama, B. (2021). Heterogeneous multiphase flow properties of volcanic rocks and implications for noble gas transport from underground nuclear explosions. Vadose Zone J, 2021;e20123. doi:10.1002/vzj2.20123

[6] Priesack, E., & Durner, W. (2006). Closed-form expression for the multimodal unsaturated conductivity function. Vadose Zone Journal, 5, 121–124. doi:10.2136/vzj2005.0066

## PE1 Experimental Team Members

1. Los Alamos National Laboratory
2. Pacific Northwest National Laboratory
3. Sandia National Laboratories
4. Lawrence Livermore National Laboratory
5. New Mexico Institute of Mining and Technology
6. Atomic Weapons Establishment
7. Nevada National Security Site
8. Mission Support and Test Services
9. University of Nevada, Reno

George Abbott^6^

Thomas Alexander^2^

Ethan Alger^4^

Adan Alvarez^7^

Tarabay Antoun^4^

Graham Auld^6^

Perry Barrow^3^

Tara Bartlett^7^

Miles Bodmer^3^

Kyren Bogolub^3^

Jesse Bonner^7^

Rose Borden^3^

Hakim Boukhalfa^1^

Chris Bradley^1^

Scott Broome^3^

Brian Brown^7^

Jeff Burghardt^2^

Charles Choens^3^

Al Churby^4^

Alexander Couture^2^

Glenn Crosby^4^

Alvaro Cruz-Cabrera^3^

Walter Dekin^4^

Matthew Dietel^7^

Christine Downs^3^

Nicholas Downs^7^

Elizabeth Dzenitis^4^

Eric Eckert^7^

Stephanie Eras^3^

Garrett Euler^1^

Souheil Ezzedine^4^

Jose Falliner^3^

Jim Fast^2^

Kristine Featherston^7^

Joshua Feldman^2^

Michael Foxe^2^

Clayton Freimuth^7^

Bradley Fritz^2^

Graham Galvin^6^

Sergio Gamboa^8^

Lisa Garner^7^

Jason Gastelum^2^

Jessie Gaylord^4^

David Gessey^7^

Matthew Goodwin^6^

James Griego^3^

Scott Grover^7^

Dylan Hauk^2^

Jason Heath^3^

Austin Holand^3^

James Holdcroft^6^

Will Honjas^9^

Matthew Ingraham^3^

Johnny Jaramillo^3^

Aryton Jenkins^6^

Kyle Jones^3^

Graham Kent^9^

Michael Keogh^7^

Will Kibikas^3^

Hunter Knox^2^

James Knox^2^

Kristopher Kuhlman^3^

Jennifer Larotonda^7^

Dorothy Linneman^2^

Paul Lipkowitz^8^

Gordon MacLeod^1^

Erin McCann^2^

Rob Mellors^4^

Brian Memmott^7^

Jennifer Mendez^2^

Xavier Miller^7^

Manny Montano^3^

Joseph Morris^4^

William Munley^2^

Dea Musa^1^

Steve Myers ^4^

Annabelle Navarro^2^

Shawn Otto^1^

Rose Perea^2^

Jacob Peterson^2^

Gabe Plank^9^

Mike Poskey^7^

Matthew Powell^3^

Amanda Price^4^

Andrew Puyleart^2^

Justin Reppart^7^

Hernan Rico^7^

Barry Roberts^3^

Rebecca Rodd^4^

Mark Rodriguez^3^

Alexander Romanczuk^4^

Melissa Roth^2^

George Salyer^7^

Bill Savran^9^

Cari Seifert^2^

Dana Sirota^2^

Dave Slater^9^

Devon Smith^7^

Ken Smith^9^

Cathy Snelson^1^

Brady Spears^1^

Philip Stauffer^1^

Richard Stead^1^

Mary Stephens^7^

Chris Strickland^2^

Joshua Tafoya^3^

M'balia Tagoe^7^

Stephanie Teich-McGoldrick^3^

Ben Terry^6^

Ryan Thompson^8^

Margaret Townsend^7^

Greg Tubbs^1^

Reagan Turley^7^

Nichole Valdez^3^

Oleg Vorobiev^4^

Robert White^7^

Aliya Whitehill^2^

Marc Williams^3^

Jennifer Wilson^3^

Lynn Wood^2^

Andrew Wright^3^

Guangping Xu^3^

Cleat Zeiler^7^

## Figures


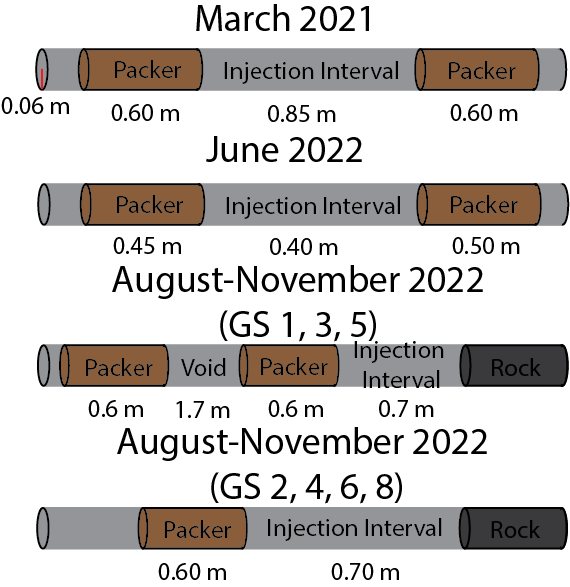


**Figure S1** Packer assembly designs performed from March 2021 to November 2022 in P-Tunnel, NNSS, NV.


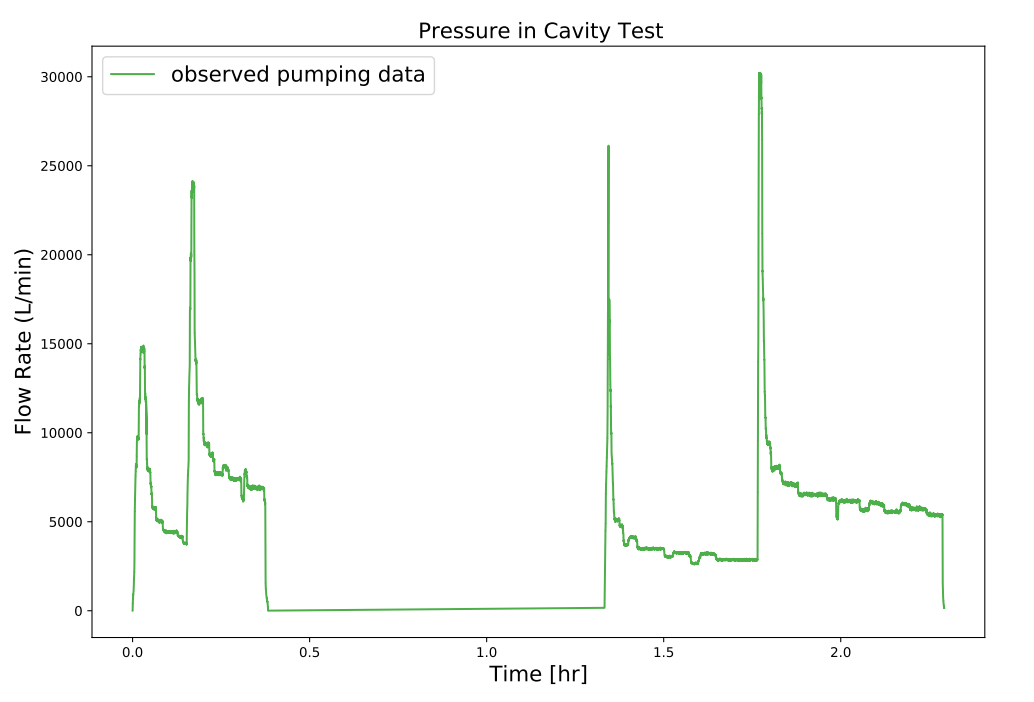


**Figure S2** Air flow rate into the cavity for the pressurization test.


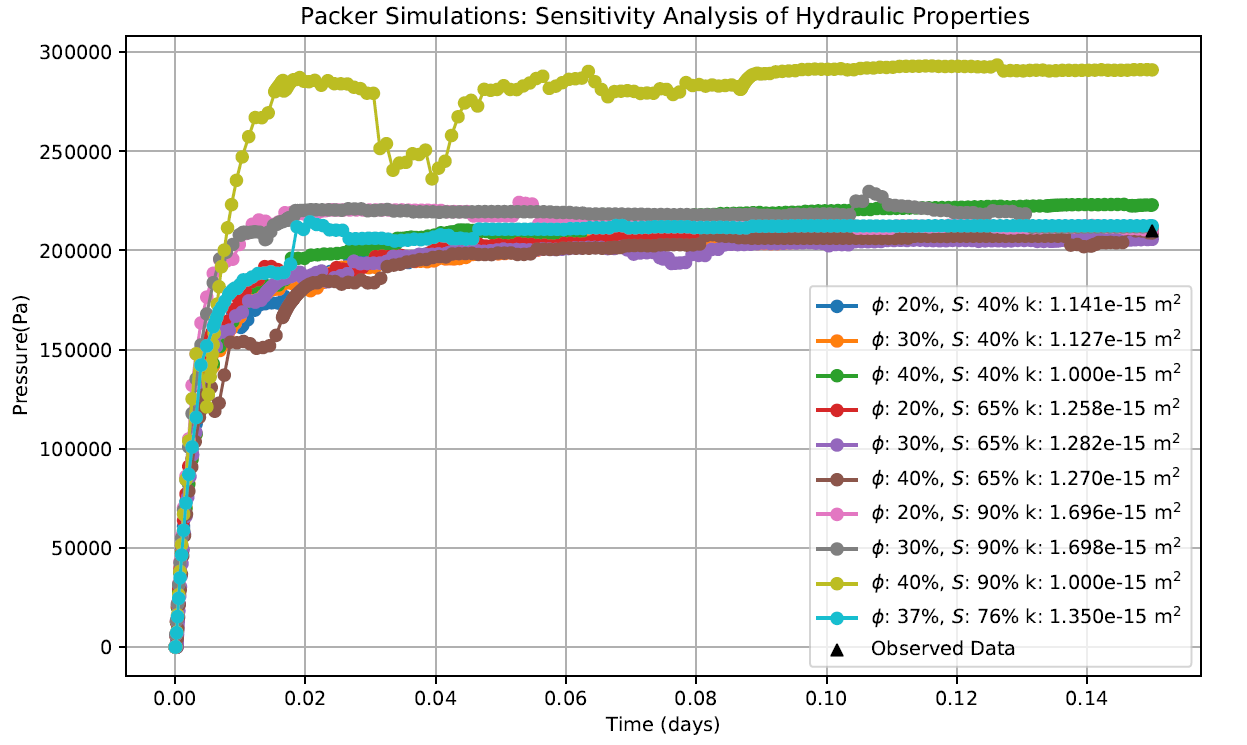


**Figure S3.** Sensitivity analysis of saturation (S) and porosity (*ɸ*). Using a range of porosity and saturation values, PEST calibrated permeability of packer test numerical models to match the observed steady state pressure (black triangle). Permeability varied from 1.000E-15 – 1.698E-15 m^2^. The average porosity and saturation are 37% and 76% respectively.


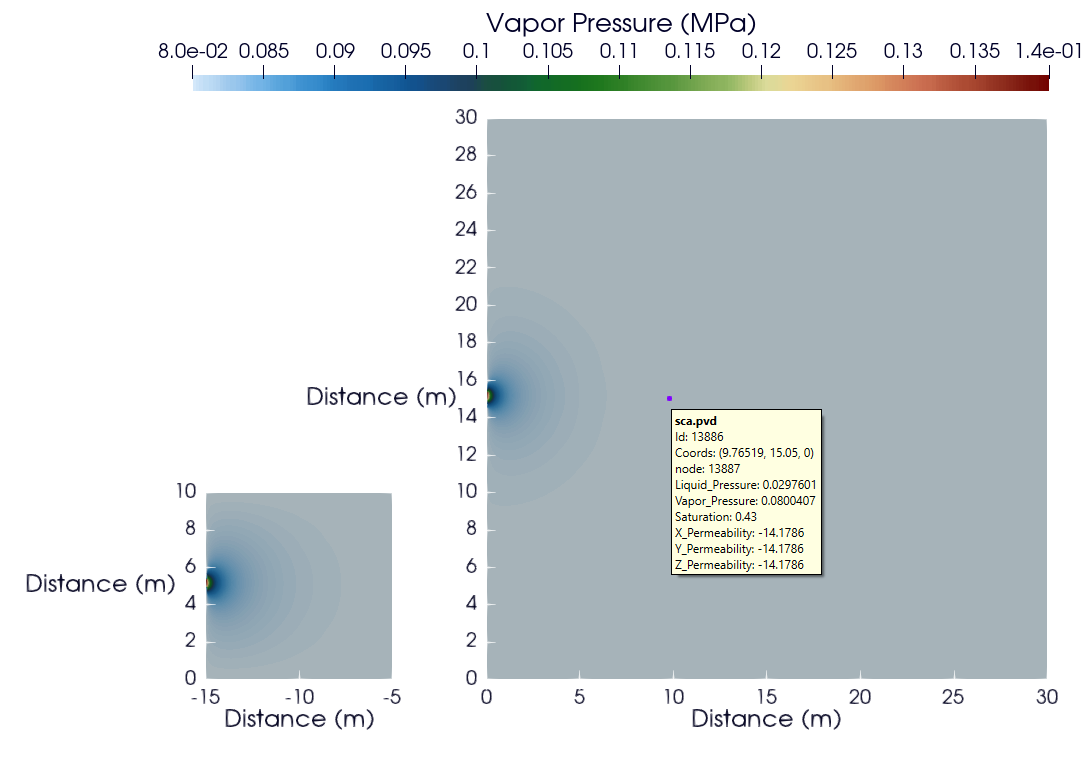


**Figure S4.** Boundary effect test performed on the original (10x10 m^2^) and larger (30x30 m^2^) mesh. For the test, the highest flow rate of 20 L/min was used. Excess pressure at the 10 m boundary in the larger mesh was 40 Pa or 0.005 psi.


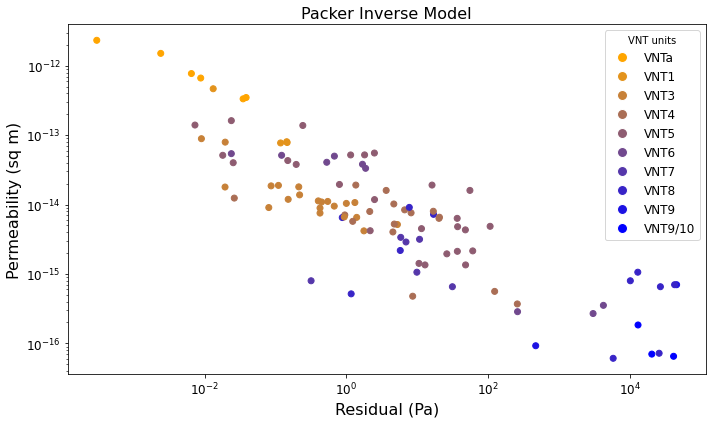


**Figure S5.** Packer test permeabilities calibrated using PEST plotted against residuals. Error (residual) increases with decreasing permeability. Colors correspond to VNT units, the shallowest is VNTa and the deepest is VNT9/10.


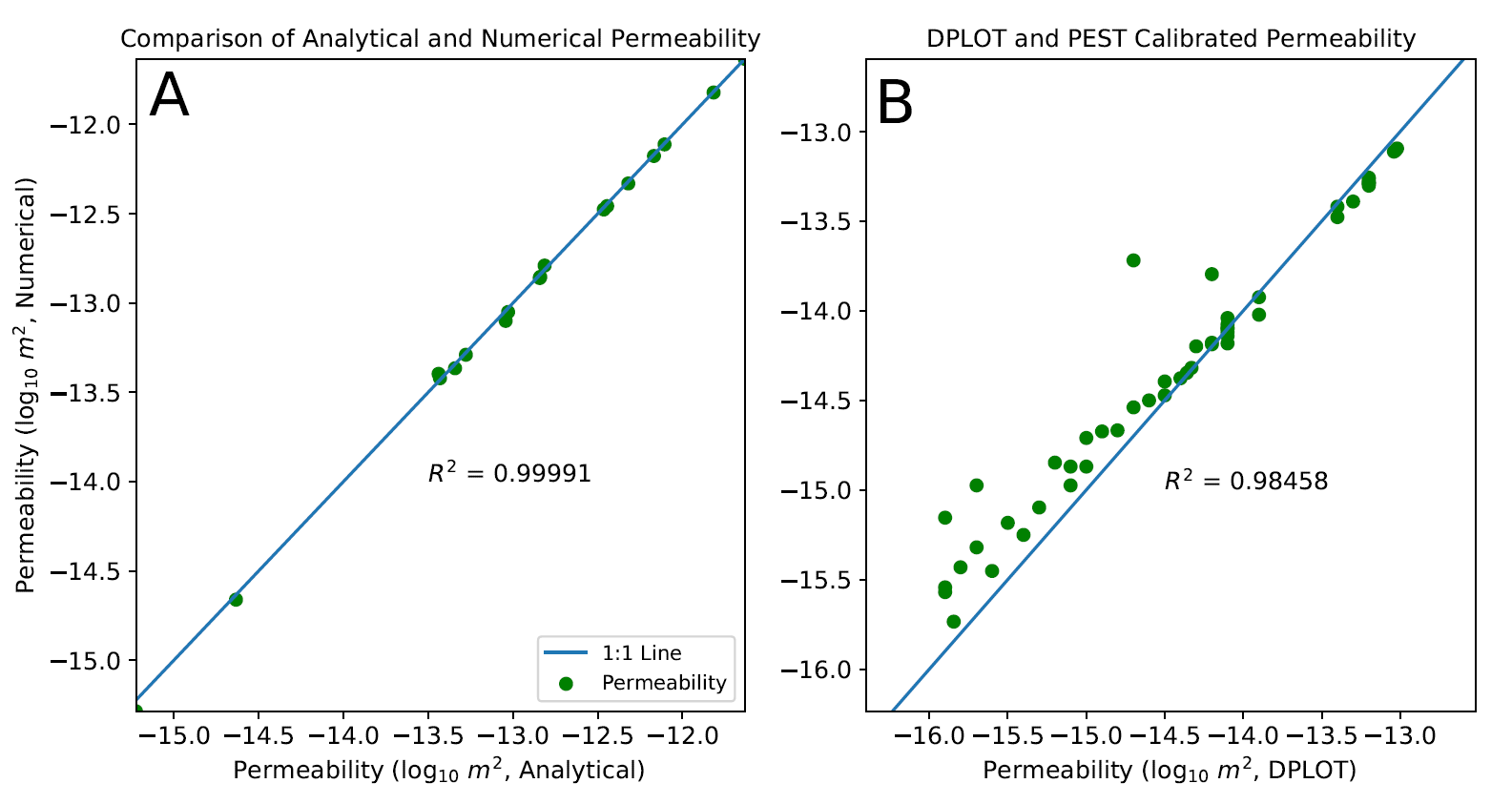


**Figure S6.** Comparison of the analytically-derived permeability versus numerically-derived permeability estimates. Analytical gas-phase permeability is calculated using Equation S17.


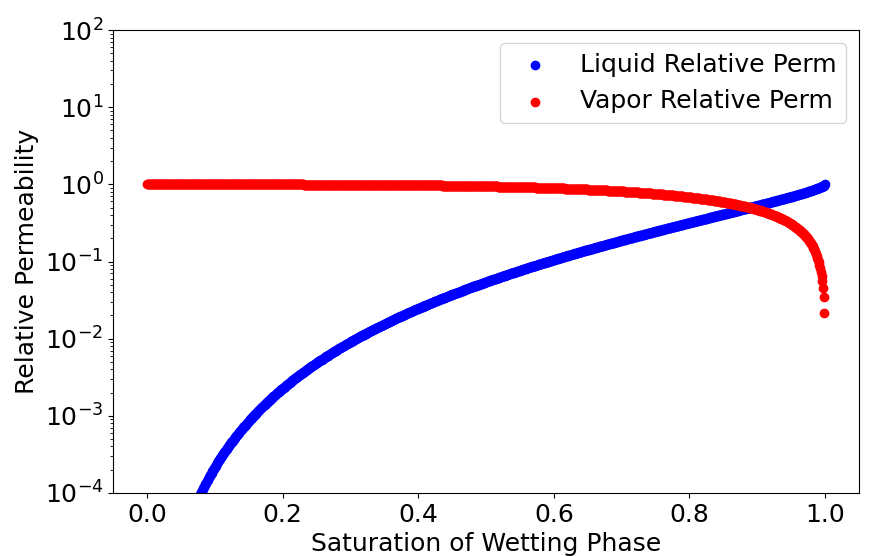


**Figure S7.** Relative permeability curves for the liquid and vapor phase from Heath et al. (2021). In the present case, the wetting phase is water.


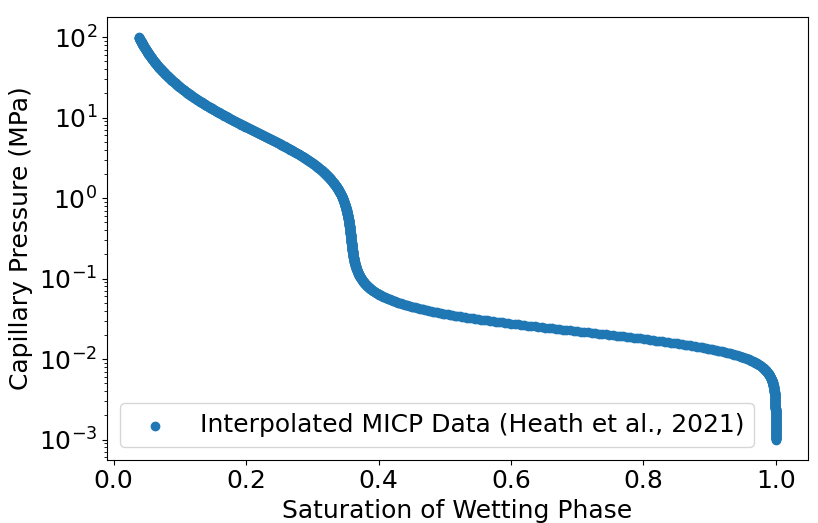


**Figure S8** Interpolated mercury intrusion data to show the multimodal nature of capillary pressure in the P-Tunnel tuffs (Heath et al., 2021).

## Tables

**Table S1.** Initial and range of permeability PEST used to calibrate the cavity pressurization test measurements for the eleven geologic units/features.

| Geologic Unit/Feature | Initial Guess  [m^2^] | Lower Limit  [m^2^] | Upper Limit [m^2^] |
| --- | --- | --- | --- |
| VNTa | 1.45E-13 | 1.45E-16 | 1.00E-11 |
| VNT1 | 4.00E-13 | 4.00E-16 | 1.00E-11 |
| VNT3 | 1.84E-12 | 1.84E-15 | 1.00E-11 |
| VNT4 | 2.31E-12 | 2.31E-15 | 1.00E-11 |
| VNT5 | 4.81E-14 | 4.81E-17 | 1.00E-11 |
| VNT6 | 7.24E-14 | 7.24E-17 | 1.00E-11 |
| VNT7 | 6.62E-14 | 6.62E-17 | 1.00E-11 |
| VNT8 | 3.44E-14 | 3.44E-17 | 1.00E-11 |
| VNT9 | 1.56E-16 | 1.56E-19 | 1.56E-13 |
| VNT10 | 6.22E-17 | 6.22E-20 | 6.22E-14 |
| Cavity | 1.00E-11 | 3.00E-15 | 1.00E-11 |

**Table S2.** Observed and PEST-calibration interval pressures. Residuals are the difference between observed and PEST-calibration values. Units for residuals are in MPa.

| **Borehole ID** | **Geologic Unit** | **Flow (L/min)** | **Interval Pressure (MPa)** | | **Residual (MPa)** | **Permeability (m^2^)** |
| --- | --- | --- | --- | --- | --- | --- |
|  |  |  | **Measured** | **FEHM/PEST** |  |  |
| GI-2 | VNT3 | 2.00 | 0.0202 | 0.0202 | 4.47E-07 | 1.09E-14 |
| GI-2 | VNT3 | 2.00 | 0.0125 | 0.0125 | -8.67E-08 | 1.86E-14 |
| GI-2 | VNT3 | 2.00 | 0.0206 | 0.0206 | 1.32E-06 | 1.07E-14 |
| GI-2 | VNT3 | 2.00 | 0.0128 | 0.0128 | 2.13E-07 | 1.80E-14 |
| GI-4 | VNT3 | 2.00 | 0.0238 | 0.0239 | 8.04E-08 | 9.06E-15 |
| GI-4 | VNT3 | 2.00 | 0.0383 | 0.0383 | -5.26E-06 | 5.16E-15 |
| GI-3 | VNT3 | 2.00 | 0.0163 | 0.0163 | 2.19E-07 | 1.38E-14 |
| GI-3 | VNT3 | 2.00 | 0.0123 | 0.0123 | 1.10E-07 | 1.89E-14 |
| GI-3 | VNT3 | 2.00 | 0.0129 | 0.0129 | 1.94E-08 | 1.79E-14 |
| GI-2 | VNT3 | 1.00 | 0.0150 | 0.0150 | -4.25E-07 | 7.54E-15 |
| GI-2 | VNT3 | 1.00 | 0.0103 | 0.0103 | -4.02E-07 | 1.13E-14 |
| GI-4 | VNT3 | 1.00 | 0.0171 | 0.0171 | 1.40E-06 | 6.54E-15 |
| GI-4 | VNT3 | 1.00 | 0.0253 | 0.0253 | 1.77E-06 | 4.18E-15 |
| GI-3 | VNT3 | 1.00 | 0.0129 | 0.0129 | -4.25E-07 | 8.92E-15 |
| GI-3 | VNT3 | 1.00 | 0.0106 | 0.0106 | -5.46E-07 | 1.11E-14 |
| GI-3 | VNT3 | 1.00 | 0.0112 | 0.0112 | -1.00E-06 | 1.04E-14 |
| GI-2 | VNT4 | 2.00 | 0.0132 | 0.0132 | 1.36E-06 | 1.91E-14 |
| GI-4 | VNT4 | 2.00 | 0.0350 | 0.0350 | -2.03E-05 | 6.33E-15 |
| GI-3 | VNT4 | 2.00 | 0.0156 | 0.0156 | -3.65E-06 | 1.60E-14 |
| GI-3 | VNT4 | 2.00 | 0.0318 | 0.0318 | -9.51E-07 | 7.09E-15 |
| GI-2 | VNT4 | 1.00 | 0.0104 | 0.0104 | -2.62E-08 | 1.24E-14 |
| GI-4 | VNT4 | 1.00 | 0.0227 | 0.0228 | -4.75E-06 | 5.24E-15 |
| GI-3 | VNT4 | 1.00 | 0.0126 | 0.0126 | 4.68E-06 | 1.02E-14 |
| GI-3 | VNT4 | 1.00 | 0.0210 | 0.0210 | -1.23E-06 | 5.73E-15 |
| GI-4 | VNT5 | 2.00 | 0.0462 | 0.0463 | -1.07E-04 | 4.86E-15 |
| GI-4 | VNT5 | 2.00 | 0.0143 | 0.0143 | 7.99E-07 | 1.95E-14 |
| GI-4 | VNT5 | 1.00 | 0.0294 | 0.0294 | 4.79E-05 | 4.32E-15 |
| GI-4 | VNT5 | 1.00 | 0.0121 | 0.0121 | -2.50E-06 | 1.18E-14 |
| AC-1 | VNT4 | 3.31 | 0.0600 | 0.0600 | -6.65E-06 | 8.39E-15 |
| AC-1 | VNT4 | 7.15 | 0.1095 | 0.1095 | -2.14E-06 | 7.95E-15 |
| AC-1 | VNT4 | 12.14 | 0.1616 | 0.1616 | -8.20E-06 | 7.60E-15 |
| AC-1 | VNT5 | 0.53 | 0.0623 | 0.0623 | 1.06E-05 | 1.42E-15 |
| AC-1 | VNT5 | 1.39 | 0.1287 | 0.1287 | -1.29E-05 | 1.35E-15 |
| AC-1 | VNT5 | 2.98 | 0.2099 | 0.2099 | -4.82E-05 | 1.35E-15 |
| AC-1 | VNT7 | 1.39 | 0.0703 | 0.0703 | -6.95E-06 | 2.89E-15 |
| AC-1 | VNT7 | 3.60 | 0.1298 | 0.1298 | 1.08E-05 | 3.16E-15 |
| AC-1 | VNT7 | 7.06 | 0.1932 | 0.1932 | 5.86E-06 | 3.37E-15 |
| AC-1 | VNT8 | 0.13 | 0.0778 | 0.0507 | 2.71E-02 | 6.55E-16 |
| AC-1 | VNT8 | 0.13 | 0.1450 | 0.1552 | -1.02E-02 | 7.98E-16 |
| AC-1 | VNT8 | 0.64 | 0.2233 | 0.2363 | -1.30E-02 | 1.06E-15 |
| AC-1 | VNT9 | 0.14 | 0.0804 | 0.0346 | 4.59E-02 | 7.00E-16 |
| AC-1 | VNT9 | 0.14 | 0.1464 | 0.1459 | 4.71E-04 | 9.24E-17 |
| AC-1 | VNT9/10 | 0.12 | 0.0701 | 0.0832 | -1.31E-02 | 1.84E-16 |
| AC-1 | VNT9/10 | 0.13 | 0.1426 | 0.1630 | -2.05E-02 | 7.00E-17 |
| AC-1 | VNT9/10 | 0.13 | 0.2194 | 0.1779 | 4.16E-02 | 6.51E-17 |
| GI-5 | VNT5 | 2.10 | 0.0688 | 0.0688 | -3.72E-05 | 4.80E-15 |
| GI-5 | VNT5 | 5.59 | 0.1427 | 0.1427 | 1.15E-05 | 4.50E-15 |
| GI-5 | VNT5 | 9.54 | 0.2097 | 0.2097 | 2.17E-06 | 4.20E-15 |
| GI-5 | VNT5 | 0.92 | 0.0741 | 0.0742 | -2.62E-05 | 1.95E-15 |
| GI-5 | VNT5 | 2.65 | 0.1456 | 0.1455 | 3.69E-05 | 2.12E-15 |
| GI-5 | VNT5 | 4.77 | 0.2099 | 0.2099 | 6.08E-05 | 2.15E-15 |
| GI-6 | VNT5 | 6.40 | 0.0225 | 0.0225 | -2.49E-06 | 5.53E-14 |
| GI-6 | VNT5 | 13.55 | 0.0437 | 0.0437 | -1.15E-06 | 5.19E-14 |
| GI-6 | VNT5 | 19.90 | 0.0593 | 0.0593 | 1.81E-06 | 5.20E-14 |
| GI-6 | VNT6 | 0.13 | 0.0721 | 0.0691 | 3.04E-03 | 2.69E-16 |
| GI-6 | VNT6 | 0.36 | 0.1396 | 0.1399 | -2.61E-04 | 2.86E-16 |
| GI-6 | VNT6 | 0.83 | 0.2139 | 0.2097 | 4.25E-03 | 3.53E-16 |
| GI-6 | VNT6 | 6.83 | 0.0352 | 0.0352 | -1.87E-06 | 3.33E-14 |
| GI-6 | VNT6 | 12.99 | 0.0528 | 0.0528 | 1.69E-06 | 3.81E-14 |
| GI-6 | VNT6 | 19.53 | 0.0686 | 0.0686 | 5.29E-07 | 4.07E-14 |
| GI-6 | VNT6 | 7.18 | 0.0261 | 0.0261 | -6.78E-07 | 4.99E-14 |
| GI-6 | VNT6 | 13.73 | 0.0435 | 0.0435 | 1.22E-07 | 5.13E-14 |
| GI-6 | VNT6 | 20.10 | 0.0563 | 0.0563 | -2.38E-08 | 5.42E-14 |
| GI-6 | VNT7 | 0.28 | 0.0643 | 0.0643 | 3.14E-05 | 6.55E-16 |
| GI-6 | VNT7 | 0.94 | 0.1331 | 0.1331 | -3.18E-07 | 7.98E-16 |
| GI-6 | VNT7 | 2.41 | 0.2048 | 0.2048 | 9.90E-06 | 1.06E-15 |
| GI-6 | VNT8 | 3.28 | 0.0559 | 0.0559 | -7.74E-06 | 9.11E-15 |
| GI-6 | VNT8 | 6.70 | 0.1121 | 0.1121 | -1.70E-05 | 7.23E-15 |
| GI-6 | VNT8 | 11.02 | 0.1683 | 0.1683 | -8.76E-07 | 6.50E-15 |
| GI-6 | VNT8 | 0.16 | 0.0823 | 0.0394 | 4.30E-02 | 7.00E-16 |
| GI-6 | VNT8 | 0.14 | 0.1437 | 0.1697 | -2.60E-02 | 7.20E-17 |
| GI-6 | VNT8 | 0.19 | 0.2180 | 0.2238 | -5.84E-03 | 6.09E-17 |
| HF-1 | VNT1 | 7.71 | 0.0190 | 0.0190 | -1.18E-07 | 7.73E-14 |
| HF-1 | VNT1 | 15.20 | 0.0334 | 0.0334 | -1.46E-07 | 7.84E-14 |
| HF-1 | VNT1 | 20.10 | 0.0411 | 0.0411 | -1.44E-07 | 8.05E-14 |
| HF-1 | VNT3 | 2.79 | 0.0601 | 0.0601 | -9.34E-07 | 6.63E-15 |
| HF-1 | VNT3 | 8.18 | 0.1026 | 0.1026 | 6.75E-07 | 9.50E-15 |
| HF-1 | VNT3 | 15.10 | 0.1342 | 0.1342 | -1.51E-07 | 1.19E-14 |
| HF-1 | VNT4 | 0.21 | 0.0808 | 0.0810 | -2.59E-04 | 3.70E-16 |
| HF-1 | VNT4 | 0.57 | 0.1339 | 0.1339 | 8.64E-06 | 4.78E-16 |
| HF-1 | VNT4 | 1.30 | 0.2070 | 0.2072 | -1.24E-04 | 5.61E-16 |
| HF-1 | VNT4 | 1.75 | 0.0650 | 0.0650 | -4.54E-06 | 4.03E-15 |
| HF-1 | VNT4 | 5.60 | 0.1057 | 0.1057 | -2.06E-05 | 6.57E-15 |
| HF-1 | VNT4 | 12.10 | 0.1557 | 0.1558 | -1.69E-05 | 8.01E-15 |
| HF-1 | VNT5 | 1.10 | 0.0549 | 0.0549 | -1.62E-05 | 1.91E-14 |
| HF-1 | VNT5 | 5.10 | 0.1197 | 0.1197 | -3.67E-05 | 6.33E-15 |
| HF-1 | VNT5 | 10.60 | 0.1683 | 0.1683 | 5.55E-05 | 1.60E-14 |
| GS-6 | VNTa | 8.31 | 0.0019 | 0.0019 | -8.80E-09 | 6.66E-13 |
| GS-4 | VNTa | 8.30 | 0.0036 | 0.0036 | -3.85E-08 | 3.49E-13 |
| GS-5 | VNT8 | 0.65 | 0.0362 | 0.0362 | -5.77E-06 | 2.18E-15 |
| GS-3 | VNT5 | 0.95 | 0.0040 | 0.0040 | 2.53E-08 | 4.01E-14 |
| GS-7 | VNT3 | 2.32 | 0.0039 | 0.0039 | -1.95E-08 | 7.94E-14 |
| GS-2 | VNT1 | 8.66 | 0.0028 | 0.0028 | 1.32E-08 | 4.67E-13 |
| GS-1 | VNT5 | 7.42 | 0.0073 | 0.0073 | -2.38E-08 | 1.62E-13 |
| GS-8 | VNTa | 7.71 | 0.0008 | 0.0008 | -2.40E-09 | 1.51E-12 |
| GS-3 | VNT5 | 4.96 | 0.0190 | 0.0190 | 1.96E-07 | 3.79E-14 |
| GS-6 | VNTa | 9.35 | 0.0019 | 0.0019 | -6.50E-09 | 7.73E-13 |
| GS-5 | VNT8 | 1.17 | 0.1588 | 0.1588 | -1.17E-06 | 5.17E-16 |
| GS-4 | VNTa | 9.38 | 0.0042 | 0.0042 | 3.49E-08 | 3.34E-13 |
| GS-3 | VNT5 | 1.88 | 0.0054 | 0.0054 | -1.80E-08 | 5.13E-14 |
| GS-3 | VNT5 | 5.58 | 0.0175 | 0.0175 | 1.49E-07 | 4.31E-14 |
| GS-1 | VNT5 | 5.50 | 0.0058 | 0.0058 | -7.30E-09 | 1.40E-13 |
| GS-7 | VNT3 | 6.10 | 0.0097 | 0.0097 | -9.00E-09 | 8.90E-14 |
| GS-8 | VNTa | 9.21 | 0.0006 | 0.0006 | -3.00E-10 | 2.33E-12 |
| GS-1 | VNT5 | 8.19 | 0.0086 | 0.0086 | 2.43E-07 | 1.38E-13 |

**Table S3** Comparison between PEST-calibrated and analytical calculated permeability.

| **Permeability (sq m) Numerical vs Analytical** | | | | |
| --- | --- | --- | --- | --- |
| **Borehole ID** | **Unit** | **Numerical** | **Analytical** | **% Error** |
| GS-6 | VNTa | 6.66E-13 | 6.79E-13 | 2% |
| GS-4 | VNTa | 3.49E-13 | 3.59E-13 | 3% |
| GS-8 | VNTa | 1.51E-12 | 1.53E-12 | 1% |
| GS-6 | VNTa | 7.73E-13 | 7.84E-13 | 1% |
| GS-4 | VNTa | 3.34E-13 | 3.43E-13 | 3% |
| GS-8 | VNTa | 2.33E-12 | 2.33E-12 | 0% |
| GS-2 | VNT1 | 4.67E-13 | 4.79E-13 | 3% |
| GS-7 | VNT3 | 7.94E-14 | 9.05E-14 | 12% |
| GS-7 | VNT3 | 8.90E-14 | 9.36E-14 | 5% |
| GS-3 | VNT5 | 4.01E-14 | 3.64E-14 | -10% |
| GS-1 | VNT5 | 1.62E-13 | 1.54E-13 | -5% |
| GS-3 | VNT5 | 3.79E-14 | 3.71E-14 | -2% |
| GS-3 | VNT5 | 5.13E-14 | 5.27E-14 | 3% |
| GS-3 | VNT5 | 4.31E-14 | 4.55E-14 | 5% |
| GS-1 | VNT5 | 1.40E-13 | 1.45E-13 | 3% |
| GS-1 | VNT5 | 1.38E-13 | 1.44E-13 | 4% |
| GS-5 | VNT8 | 2.18E-15 | 2.32E-15 | 6% |
| GS-5 | VNT8 | 5.17E-16 | 5.96E-16 | 13% |

**Table S4** Van Genutchen properties of VNT layers from Heath et al. (2021).

| **Borehole** | $\boldsymbol{log(}\boldsymbol{\alpha}_{\boldsymbol{0}}\boldsymbol{)}$ | $\boldsymbol{m}_{\boldsymbol{0}}$ | $\boldsymbol{log(}\boldsymbol{\alpha}_{\boldsymbol{1}}\boldsymbol{)}$ | $\boldsymbol{m}_{\boldsymbol{1}}$ | $\boldsymbol{w}_{\boldsymbol{0}}$ |
| --- | --- | --- | --- | --- | --- |
| VNT a-10 | 4.297 | 0.700 | 6.585 | 0.408 | 0.640 |
